# Presence of the Hmq System and Production of 4-Hydroxy-3-Methyl-2-Alkylquinolines Are Heterogeneously Distributed between Burkholderia cepacia Complex Species and More Prevalent among Environmental than Clinical Isolates

**DOI:** 10.1128/spectrum.00127-21

**Published:** 2021-06-16

**Authors:** Pauline M. L. Coulon, James E. A. Zlosnik, Eric Déziel

**Affiliations:** a Centre Armand-Frappier Santé Biotechnologie, Institut National de la Recherche Scientifique, Laval, Quebec, Canada; b Canadian Burkholderia cepacia Complex Research and Referral Repository, University of British Columbia, Vancouver, British Columbia, Canada; University of Minnesota

**Keywords:** *hmqABCDEFG* operon, 4-hydroxy-2-alkylquinolines, *pqsABCDE*, *Pseudomonas* quinolone signal, plant growth promotion, quorum sensing, secondary metabolites

## Abstract

The Burkholderia cepacia complex (Bcc) comprises several species of closely related, versatile bacteria. Some Bcc strains produce 4-hydroxy-3-methyl-2-alkylquinolines (HMAQs), analogous to the 4-hydroxy-2-alkylquinolines of Pseudomonas aeruginosa. Using *in silico* analyses, we previously estimated that the *hmqABCDEFG* operon, which encodes enzymes involved in the biosynthesis of HMAQs, is carried by about one-third of Bcc strains, with considerable inter- and intraspecies variability. In the present study, we investigated by PCR, using consensus primers, the distribution of *hmqABCDEFG* in a collection of 312 Bcc strains (222 of clinical and 90 of environmental origins) belonging to 18 Bcc species. We confirmed that this operon is not distributed evenly among Bcc species. Among the 30% of strains bearing the *hmqABCDEFG* operon, we found that 92% of environmental isolates and 82% of clinically isolated Bcc strains produce levels of HMAQs detectable by liquid chromatography-mass spectrometry in at least one of the tested culture conditions. Among the *hmqABCDEFG*-positive but HMAQ-negative strains, none expressed the *hmqA* gene under the specified culture conditions. Interestingly, the *hmqABCDEFG* operon is more prevalent among plant root environment species (e.g., Burkholderia ambifaria and Burkholderia cepacia) and absent in species commonly found in chronically colonized individuals with cystic fibrosis (e.g., Burkholderia cenocepacia and Burkholderia multivorans), suggesting a role for the Hmq system in niche adaptation. We investigated the impact of the Hmq system on plant growth promotion and found that Pisum sativum root development by *B. ambifaria* required a functional HMAQ system.

**IMPORTANCE** Environmental bacteria belonging to the various closely related species forming the Burkholderia cepacia complex (Bcc) can infect plants and animals, including humans. Their pathogenicity is regulated by intercellular communication, or quorum sensing, allowing them to collaborate instead of acting individually. Bcc organisms generally exploit interacting quorum sensing systems based on *N*-acyl-homoserine lactones as signaling molecules. Several Bcc strains also carry an *hmqABCDEFG* operon responsible for the biosynthesis of 4-hydroxy-3-methyl-2-alkylquinolines (HMAQs), molecules analogous to the Pseudomonas quinolone signal (PQS) system of P. aeruginosa. Our finding that the prevalences of the Hmq system and HMAQ production are very different between various Bcc species suggests a key role in niche adaptation or pathogenicity. This is supported by a significant reduction in plant growth promotion in the absence of HMAQ production for a beneficial Bcc strain.

## INTRODUCTION

The environmental species of *Burkholderia* can be divided into two phylogenetic groups: (i) pathogenic species and (ii) plant-beneficial species (1). Based on this still-controversial separation (2), the latter group was reclassified as *Paraburkholderia*, based on lower GC content and lack of virulence in Caenorhabditis elegans (3, 4). The pathogenic *Burkholderia* group comprises (i) plant pathogens (e.g., Burkholderia andropogonis, which causes leaf streak on sorghum [5], and B. glumae, which causes bacterial panicle blight on rice [6–8]); (ii) the pseudomallei group, composed of B. pseudomallei (the causative agent of melioidosis), B. thailandensis (closely related to B. pseudomallei but avirulent), and B. mallei (causing glanders in equids) (9–11); and (iii) the Burkholderia cepacia complex (Bcc), comprising at least 26 different species (12–14; reviewed in reference 1), most considered opportunistic pathogens.

Bcc bacteria have been used (i) in agriculture for the biocontrol of phytopathogens and for their plant growth-promoting properties (e.g., pea protection by B. ambifaria against *Pythium* and *Aphanomyces* [15, 16]) and (ii) in bioremediation (e.g., B. vietnamiensis with its trichloroethylene degradation abilities [17, 18; reviewed in reference 19]). Bcc bacteria are also well known for secondary-metabolite production, including antibiotics (reviewed in reference [20]). However, since the 1980s, Bcc opportunistic pathogens have emerged as a serious threat among certain immunocompromised individuals (e.g., those with chronic granulomatous disease [CGD]) and people with cystic fibrosis (CF), causing the cepacia syndrome and pushing authorities to prohibit their use in biotechnological applications. It is now generally accepted that their high transmissibility and intrinsic resistance to clinically relevant antibiotics make them particularly problematic (21–23).

Cell-to-cell communication mechanisms, e.g., quorum sensing, act by (i) controlling gene transcription at the population level (24), (ii) promoting colonization, and (iii) optimizing interaction with hosts and increasing resistance to stress (25, 26). Depending on the Bcc species, at least two Cep-like quorum sensing systems may be present, synthesizing different acyl-homoserine lactones (AHLs) as ligands and regulating each other and genes implicated in virulence (27–31).

The bacterium Pseudomonas aeruginosa carries a quorum sensing system based on signaling using 4-hydroxy-2-alkylquinolines (HAQs), such as the Pseudomonas quinolone signal (PQS) and 4-hydroxy-2-heptylquinoline (HHQ) (32–34). Interestingly, some species of the Bcc (*B. ambifaria* and B. cepacia), as well as B. pseudomallei and B. thailandensis, produce analogous molecules called 4-hydroxy-3-methyl-2-alkylquinolines (HMAQs) (35–37) that are synthesized by enzymes encoded by the *hmqABCDEFG* operon (36). In contrast with P. aeruginosa HAQs, the main HMAQs produced by *Burkholderia* bear a methyl group at the 3′ position and lack saturation of their alkyl side chain. Although an increasing number of Bcc strains are being reported to produce some HMAQ congeners (36, 38–44), this remains mostly anecdotal. In contrast with the HAQ/PQS quorum sensing system of P. aeruginosa, the *Burkholderia* Hmq system does not appear to form a quorum sensing system *per se* (specific transcriptional regulation of target genes in response to concentration of signaling molecules), and instead, we and Le Guillouzer have shown that it is closely interrelated with the Cep quorum sensing system in *B. ambifaria* HSJ1 and with the three Bta quorum sensing systems in B. thailandensis E264 (36, 45, 46), as HMAQs impact AHL production in both species (36, 45, 46).

Functions of P. aeruginosa HHQ and PQS as quorum sensing autoinducers, immunomodulators, and antimicrobials have been described (47). Several studies report novel molecules belonging to the HAQ family and various bacterial species having antimicrobial activities (48–52). Apart from some intra- and interspecies activity as signals (36, 45, 46) and as low-activity antimicrobials, the primary function of HMAQs remains enigmatic (38–41, 53, 54). Nonetheless, given the demonstrated role of HAQs and PQS in P. aeruginosa, HMAQs may also play roles in niche competition and virulence of producing *Burkholderia* strains (36, 46). We previously characterized a series of clinical *B. ambifaria* strains able to produce HMAQs (36, 43). Their respective colony morphotype variant had lost the ability to produce several secondary metabolites, including HMAQs, similarly to a set of environmental *B. ambifaria* isolates (36, 43). Therefore, to better understand HMAQs, we postulated that (i) the *hmqABCDEFG* operon is more frequently found among clinical Bcc strains and (ii) among HMAQ-producing Bcc isolates, clinical strains produce higher concentrations than environmental ones. Our previous bioinformatic study on the distribution of the *hmqABCDEFG* operon in the Bcc, based on available whole-genome sequences for 1,257 strains belonging to 21 Bcc species, showed that *B. ambifaria*, B. cepacia, B. contaminans, B. pyrrocinia, B. stagnalis, B. territorii, and B. ubonensis strains carry the *hmqABCDEFG* operon, although not all strains within a species do (55). On the other hand, all sequenced genomes of B. anthina, B. arboris, B. cenocepacia, B. diffusa, B. latens, B. metallica, B. multivorans, B. pseudomultivorans, B. seminalis, B. stabilis, and B. vietnamiensis—species mainly isolates from clinical cases—that have been investigated are lacking the *hmqABCDEFG* operon (55). To experimentally validate our *in silico* study and verify the ability of Bcc isolates carrying the *hmqABCDEFG* operon to actually produce HMAQs, a collection of 312 Bcc strains, comprising 222 clinical and 90 environmental isolates belonging to 18 different Bcc species, was analyzed. We first assessed the presence of the *hmqABCDEFG* operon in their genomes and then directly determined, by liquid chromatography coupled to mass spectrometry (LC-MS), the ability of all the strains bearing the *hmqABCDEFG* operon to produce HMAQs. Our data show that the Hmq system is heterogeneously distributed between Bcc species, with high prevalence in some species (e.g., B. cepacia) and near absence in others (e.g., B. cenocepacia and *B. multivorans*). Finally, we investigated the impact of the Hmq system on plant growth using *hmqA* and *hmqG* mutants and found that Pisum sativum root growth promotion by a *B. ambifaria* strain was lost in the absence of HMAQ production.

## RESULTS

### The *hmqABCDEFG* operon is heterogeneously distributed across and within Bcc species.

**FIG 1:**
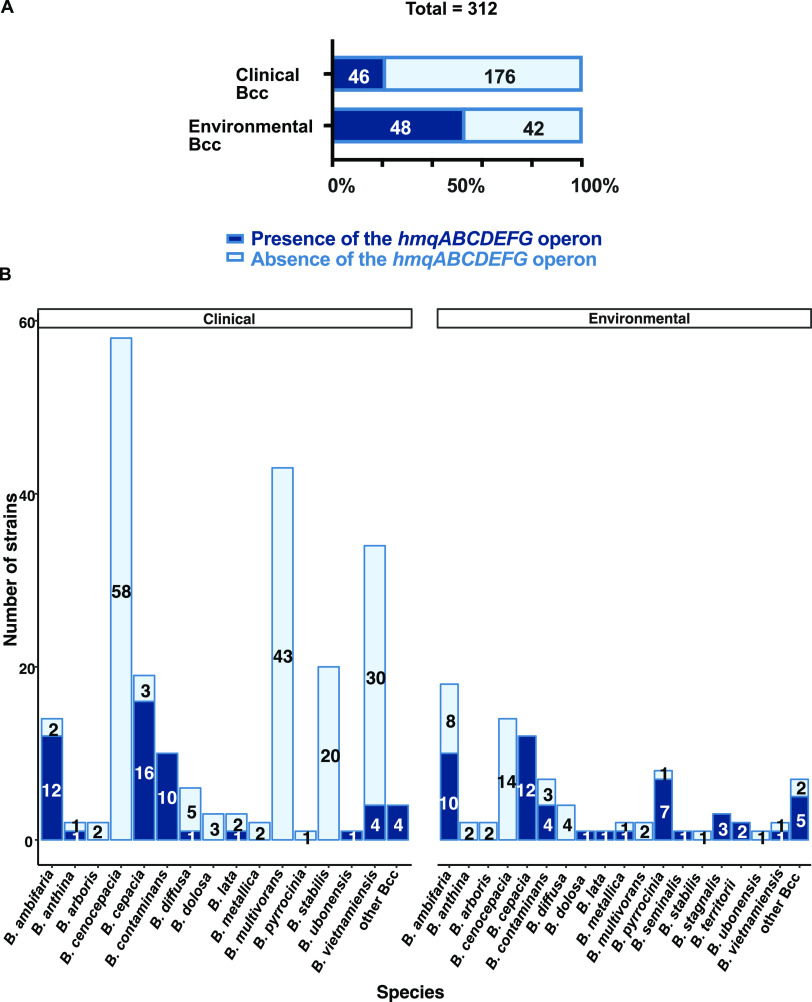
Bcc strain screening for presence of the *hmqABCDEFG* operon in their genomes. (A) Distribution of the operon between environmental and clinical Bcc strains investigated in this study (B) Distribution of the *hmqABCDEFG* operon within tested Bcc species.

We previously examined 1,257 whole-genome sequences belonging to 21 different Bcc species to assess the distribution of the *hmqABCDEFG* operon by orthology and homology analyses (55). We found that at least one sequenced strain belonging to 7 of the 21 Bcc species carries the *hmqABCDEFG* operon (*B. ambifaria*, B. cepacia, *B. contaminans B. pyrrocinia*, *B. stagnalis. B. territorii*, and *B. ubonensis*); one striking initial finding was that prevalence of the *hmqABCDEFG* operon within a species appeared highly variable (55). Here, to validate our *in silico* analyses (55) and to globally determine the ability of Bcc to actually produce HMAQs, we screened a collection of 312 Bcc strains (222 of clinical and 90 of environmental origins; listed in Table S1), belonging to 18 Bcc species: *B. ambifaria*, *B. anthina*, *B. arboris*, B. cenocepacia, B. cepacia, *B. contaminans*, *B. diffusa*, B. dolosa, B. lata, *B. metallica*, *B. multivorans*, *B. pyrrocinia*, *B. seminalis*, *B. stabilis*, *B. stagnalis. B. territorii*, *B. ubonensis*, and *B. vietnamiensis*, plus a few more classified in the “other Bcc” group (PubMLST database [https://pubmlst.org/organisms/burkholderia-cepacia-complex]). The presence of *hmqABCDEFG* was determined by PCR using consensus primers targeting *hmqA* and *hmqG* (based on highly conserved regions [Fig. S1]). We had previously shown that presence of a *hmqG* orthologue correlates with the presence of a complete *hmqABCDEFG* operon (55). Here, we found that 30% of the 312 tested strains possess an *hmqABCDEFG* operon, including 53% of the environmental strains but only 21% of clinical strains (Fig. 1A). A Fisher test with a *P* value of 2.13 × 10^−10^ indicates that the prevalence of *hmqABCDEFG* is significantly different depending on the strains’ sampling origin (environmental versus clinical). Among the 18 different Bcc species investigated, 14 species comprise at least one strain carrying the operon (Fig. 1B and Fig. S2). However, B. cenocepacia, *B. multivorans*, and *B. vietnamiensis* are overrepresented among clinical strains compared to the other species. We thus decided to subsample these three species with only 20 clinical strains, increasing the prevalence of the *hmqABCDEFG* operon in clinical Bcc strains to 30% (46 strains carrying the operon among the 151 subsampled clinical strains), which is still statistically significantly lower than the prevalence of the *hmqABCDEFG* operon within environmental strains (Fisher test with a *P* value of 1.324 × 10^−6^).

More precisely, the *hmqABCDEFG* operon is more prevalent among clinical strains of *B. ambifaria*, *B. anthina*, *B. contaminans*, *B. diffusa*, *B. ubonensis*, and *B. vietnamiensis*, while it is more prevalent among environmental strains of *B. cepacia, B. contaminans*, *B. dolosa*, *B. lata*, *B. metallica*, *B. pyrrocinia*, and the “other Bcc” group. Clinical *B. seminalis* and environmental *B. stagnalis* and *B. territorii* species carry the *hmqABCDEFG* operon.

We found isolates of *B. anthina, B. dolosa*, and *B. vietnamiensis* carrying the *hmqABCDEFG* operon, which was not predicted in our previous analysis of available genomic data (55); we confirmed these PCR results by sequencing the amplicons using primers listed in Table S2.

### Considering that horizontal gene transfer and pc3 chromosomal rearrangement could explain the heterogeneous distribution of the *hmqABCDEFG* operon in the Bcc.

Since not all Bcc species carry *hmqABCDEFG*, we asked whether the distribution of the operon could be related to a loss of the third chromosome in Bcc or to horizontal gene transfer. Bcc bacteria can lose their third chromosome, also described as a virulence megaplasmid (pc3) containing a few core genes (56, 57). The *hmqABCDEFG* operon being generally located on the pc3 replicon (except in *B. ubonensis*, which carries this operon on its second chromosome like B. pseudomallei and B. thailandensis), and because the synteny of the *hmqABCDEFG* operon is conserved within a species (55), we determined whether the strains missing the operon were also missing their pc3 replicon. Our analysis showed that the absence of the *hmqABCDEFG* operon does not correlate with the loss of pc3 (Table S3).

**FIG 2:**
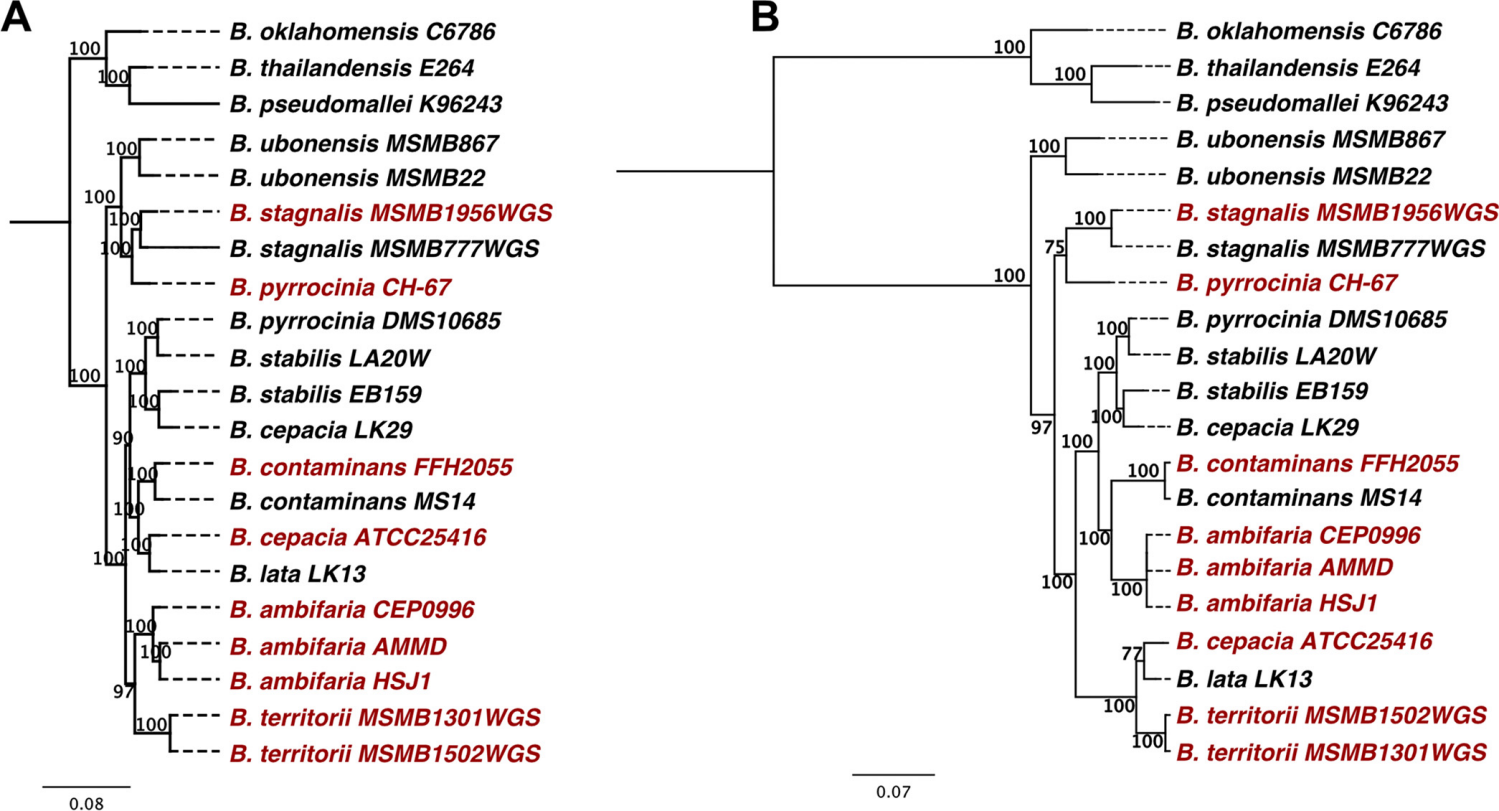
Phylogeny indicates that acquisition of the *hmqABCDEFG* operon results from horizontal gene transfer within Bcc species. (A) Phylogeny of Bcc strains carrying the *hmqABCDEFG* operon, based on MLST genes (*atpD*, *gltB*, *gyrB*, *recA*, *lepA*, *phaC*, and *trpB*) (8). (B) Phylogeny of the Bcc species based on their *hmqABCDEFG* operon. Trees were generated by RAxML using the GTRGAMMA model and 1,000 bootstraps. The branches are labeled where bootstrap values are >50%. Strains in black were chosen as models and were not a part of our study. Strains used in this study are in red.

By comparing the phylogenetic tree based on multilocus sequence typing (MLST) sequences and the distribution of strains possessing the *hmqABCDEFG* operon, we found that the resulting MLST and *hmqABCDEFG* phylogenies mostly match, except for two groups, which are inverted (Fig. 2). Hence, the Hmq system originates from a common ancestor, with possible chromosomic rearrangement for some strains.

### Most Bcc strains carrying the *hmqABCDEFG* operon produce HMAQs.

We then verified whether the presence of the biosynthetic genes is indicative of known HMAQ production. We cultured the 94 strains that were PCR-positive for *hmqA* and *hmqG* in tryptic soy broth (TSB) medium at 30°C and 60 rpm overnight and detected the production of HMAQ in 72% of the PCR-positive strains—65% clinical and 79% environmental—carrying the *hmqABCDEFG* operon (Table S4). None of the PCR-positive *B. anthina*, *B. diffusa*, *B. dolosa*, *B. metallica*, *B. seminalis*, and *B. ubonensis* strains produced detectable HMAQ under these culture conditions.

**FIG 3:**
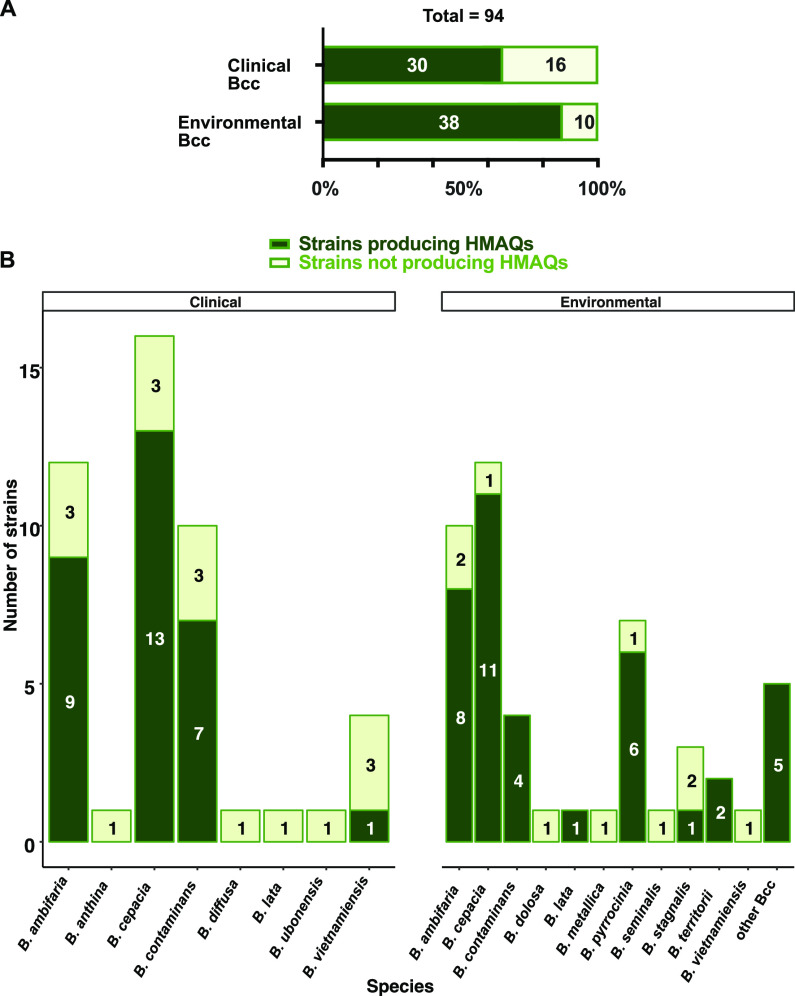
Distribution of HMAQ production among Bcc strains carrying the *hmqABCDEFG* operon. (A) Distribution of environmental and clinical Bcc strains regarding their ability to produce HMAQs in at least one of the tested culture conditions. (B) Distribution of HMAQ-producing Bcc species. HMAQs were detected and quantified by LC-MS with a limit of detection of 50 μg/liter for each molecule in the total culture.

Assuming that the absence of HMAQ production in 26 of the 94 PCR-positive strains could simply be the result of unfavorable culture conditions, we assayed the production of these metabolites in TSB but at 37°C, in artificial sputum medium (ASM; at 30°C at 60 rpm, overnight), and on tryptic soy agar plates (TSA; incubated at 30°C for 4 days). As shown in Table 1, 4 of 26 previously negative strains produced HMAQs when grown in TSB at 37°C, while ASM growth induced production levels allowing detection of HMAQs in 10 of the 26 Bcc strains—seven environmental and three clinical strains. Surface growth on TSA plates induced the detectable production of HMAQs for five environmental and four clinical strains. Among the strains already producing HMAQs in TSB at 30°C, most of them also produced HMAQs in ASM and TSA (Table S4). Interestingly, two *B. contaminans* strains that were negative in both TSB and ASM produced detectable HMAQ when cultured on TSA plates. These additional culture conditions reduced the number of HMAQ-negative strains from 26 to 15, increasing the number of Bcc isolates able to produce HMAQs to a total of 82, that is, 87% of strains carrying the *hmqABCDEFG* operon, including 92% environmental and 82% clinical isolates (Fig. 3 and Fig. S3).

**TABLE 1:** Production of HMAQs under alternative culture conditions for *hmqABCDEFG*-positive strains not producing HMAQs in tryptic soy broth at 30°C

Strain	Type	HMAQ production		
		TSB (37°C)	ASM (30°C)	TSA (30°C)
*B. ambifaria* AMMD	Environmental	−	+	+
*B. ambifaria* AU7994	Clinical	−	−	−
*B. ambifaria* CEP1231	Clinical	−	+	−
*B. ambifaria* HI2425	Environmental	−	+	+
*B. ambifaria* VC11631	Clinical	−	−	−
*B. anthina* VC15382	Clinical	−	−	−
B. cepacia ATCC 25416	Environmental	−	−	−
B. cepacia VC13196	Clinical	−	−	−
B. cepacia VC13575	Clinical	+	−	−
B. cepacia VC19225	Clinical	+	+	+
*B. contaminans* VC15406	Clinical	−	−	+
*B. contaminans* VC16897	Clinical	+	−	−
*B. contaminans* VC16948	Clinical	+	−	+
*B. diffusa* VC14008	Clinical	−	−	−
*B. dolosa* LMG21443	Environmental	−	−	−
*B. lata* VC6377	Clinical	−	+	+
*B. metallica* ES0559	Environmental	−	−	−
*B. pyrrocinia* Bcc indeterminate 9 ES0209	Environmental	+	+	+
*B. seminalis* HI2490	Environmental	−	+	−
*B. stagnalis* Bcc indeterminate 6 HI3537	Environmental	−	+	+
*B. stagnalis* HI2720	Environmental	−	+	−
*B. ubonensis* LMG24263	Clinical	−	−	−
*B. vietnamiensis* CEP0040	Clinical	−	−	−
*B. vietnamiensis* VC17180	Clinical	+	−	−
*B. vietnamiensis* VC9237	Clinical	−	−	−

To validate our strategy, we assessed the production of HMAQs by 31 Bcc strains determined by PCR not to carry the *hmqABCDEFG* operon; all 31 strains—belonging to *B. ambifaria*, *B. anthina*, *B. arboris*, B. cenocepacia, *B. multivorans*, *B. pyrrocinia*, *B. stabilis*, *B. ubonensis*, and *B. vietnamiensis*—indeed did not produce detectable HMAQs (Table S5).

### All HMAQ-producing Bcc strains mainly produce the HMAQ-C_7_:2′ and HMAQ-C9:2′ congeners.

**FIG 4:**
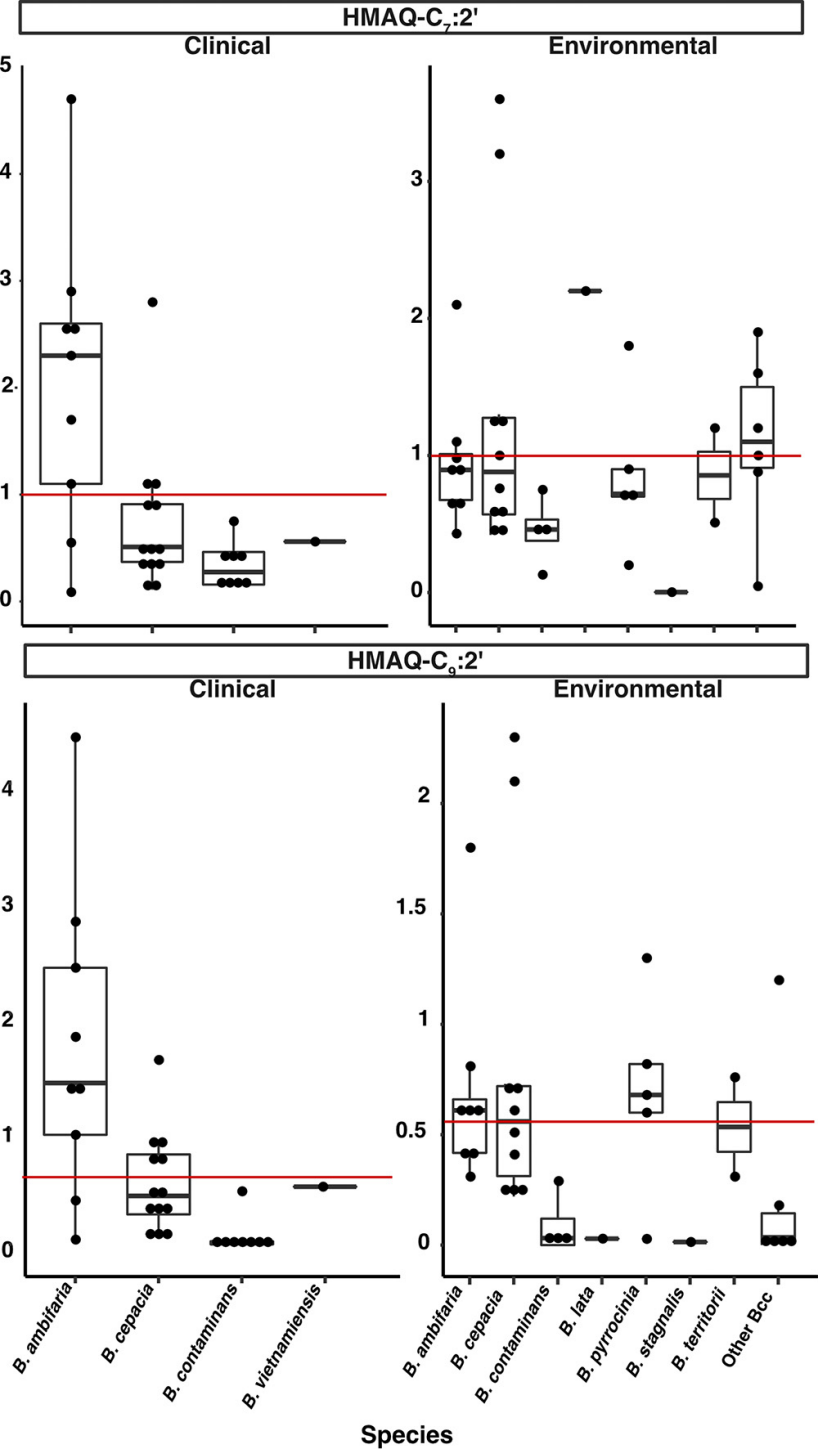
Concentration of the main produced HMAQs among Bcc species when cultured in tryptic soy broth. HMAQ-C_7_:2′ and HMAQ-C_9_:2′ were quantified by LC-MS with a lower limit of detection of 50 μg/liter for each molecule in the total culture. Tested strains are grouped by species. The production of HMAQs was quantified in three biological replicate cultures for each strain, and each dot represents the average HMAQ production for each strain, in milligrams per liter. Lines represent the production average for the clinical and environmental strains.

To verify which HMAQ congeners are produced by the various Bcc, we scanned by LC-MS for the 14 congeners of HAQs and HMAQs we had previously identified (Table S6) (36). We found that the main congeners produced were HMAQ-C_7_:2′ and HMAQ-C_9_:2′, as previously observed for *B. ambifaria* HSJ1 (36), followed by HMAQ-C_8_ and HMAQ-C_6_ (Fig. 4; Table S6). Most of the strains produced other HMAQs, such as HMAQ-C_7_ and HMAQ-C_8_:2′ (also known as burkholone).

We also found that the concentration of HMAQs produced was variable among the various species when grown in TSB at 30°C. A Kruskal-Wallis test confirmed the absence of statistical difference in the concentrations produced between the clinical and environmental strains (*P* value of 0.19 for HMAQ-C_7_:2′ and 0.22 for HMAQ-C_9_:2′). However, clinical strains of *B. ambifaria* produced more HMAQs than environmental ones. The opposite was observed for B. cepacia and *B. contaminans* strains (Fig. 4).

### Presence of the *hmqABCDEFG* operon and ability to produce HMAQs do not correlate with P. aeruginosa coisolation or the origin of the strain.

Since the *hmqABCDEFG* operon is homologous to the *pqsABCDE* operon in P. aeruginosa, and HMAQs might interact with the PQS system, we asked if HMAQ production of clinical Bcc is correlated with a coisolation or a colocalization with P. aeruginosa from the patient at some point as well as with the origin of the sample (sputum, throat, sinus, etc.). Information was available for 53 of 222 clinical strains (Table S7). Using Fisher's exact test for count data, we did not find a correlation between the presence of the *hmqABCDEFG* operon and the presence of P. aeruginosa at the sampling time or within the previous year or with the origin of the sample (Table 2).

**TABLE 2:** Correlation between the presence of the *hmqABCDEFG* operon and the production of HMAQs in clinical Bcc, along with sampling data

Characteristic	Correlation (*P*) with^*a*^:		
	Coisolation with P. aeruginosa	Colocalization with P. aeruginosa (previous yr)	Origin of sample^*b*^
Presence of the *hmqABCDEFG* operon	0.34	0.41	0.11
Production of HMAQs	0.57	1	0.59

a Raw data are from Table S7. *P* values are representative of the correlation (tested by Fisher’s exact test for count data) and are considered significant under 0.05.

b Possible origins: sputum, respiratory sample, throat, or sinus.

### Lack of expression of the *hmqABCDEFG* operon explains the absence of HMAQs detection in some Bcc cultures.

Our screening revealed that 26 strains carrying the *hmqABCDEFG* operon do not produce HMAQs in TSB at 30°C. To investigate the possibility that too low transcription of the biosynthetic genes would explain this absence of detectable production, which is compatible with the induction seen when the culture conditions were changed, we measured the transcription of the *hmqABCDEFG* operon for one HMAQ-negative and one HMAQ-positive strain per species of *B. ambifaria*, B. cepacia, *B. contaminans*, and *B. vietnamiensis* by targeting the *hmqA* gene by reverse transcription-PCR (RT-PCR).

Results show that the *hmqABCDEFG*-positive but HMAQ-negative strains *B. ambifaria* AMMD, B. cepacia ATCC 25416, *B. contaminans* VC15406, and *B. vietnamiensis* VC9237 do not express the *hmqA* gene when grown in TSB at 30°C, while *B. ambifaria* HSJ1, B. cepacia VC13394, *B. contaminans* FFH2055, and *B. vietnamiensis* VC8245, which produce HMAQs under these conditions, produce a clear *hmqA* transcript (Fig. S5). Thus, we decided to investigate if a mutation in the promoter region could explain the lack of expression of the *hmqABCDEFG* operon, but there was no difference between *B. ambifaria* HSJ1 and AMMD promoter region sequences. The lack of expression of *hmqABCDEFG* is a matter of regulation, especially since strain AMMD produced HMAQs in ASM and TSA. Extending these results, we hypothesize that the other strains which carry the *hmqABCDEFG* operon and do not produce HMAQs do not express the *hmqA* gene under the specified culture conditions. This is supported by the finding that several of these strains could eventually produce measurable levels of HMAQs when culture conditions were changed (as described above) (Table 1).

### The capacity to produce HMAQs is required for root growth promotion.

**FIG 5:**
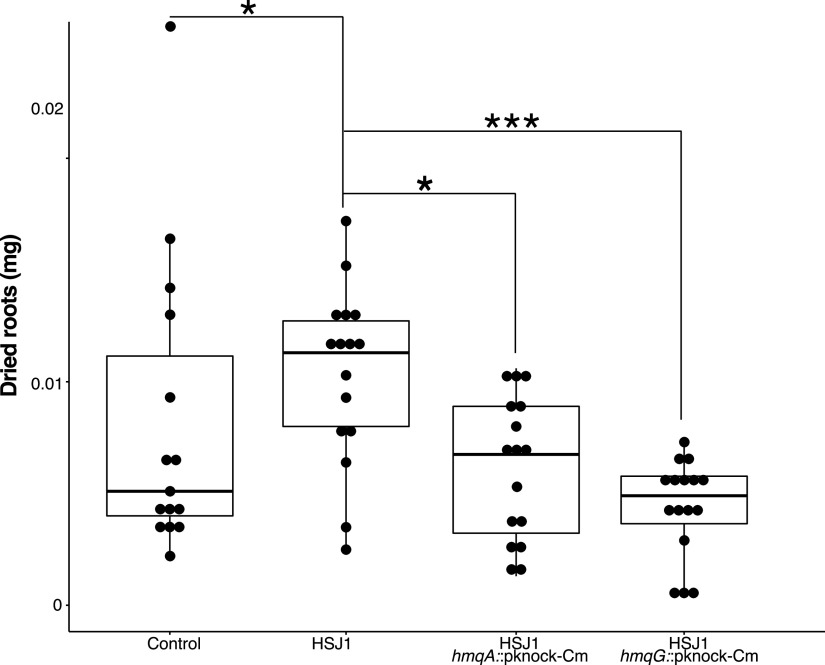
Presence of the intact *hmqABCDEFG* operon in *B. ambifaria* promotes the development of roots in a *P. sativum* plant model. The impact of *hmqA* and *hmqG* mutants of *B. ambifaria* HSJ1 on root development of *P. sativum* compared to that of the wild-type strain was measured. Weight of dried roots was measured after 5 days of cultivation. A Dunn test was performed, and *P* values are represented as follows: *, between 0.5 and 0.01; **, between 0.01 and 0.001; ***, between 0.001 and 0.0001.

Since *B. ambifaria* is a good plant growth promoter (15, 16; reviewed in reference 19) and the Hmq system is more prevalent among environmental strains, we investigated the impact of the Hmq system on growth of the common pea (*P. sativum*). While *B. ambifaria* HSJ1 promoted *P. sativum* roots’ development, the isogenic *hmqA*::pKnock-Cm and *hmqG*::pKnock-Cm mutants lost this effect (Fig. 5).

## DISCUSSION

This study aimed at understanding the prevalence of the Hmq system and corresponding HMAQ production among the various species belonging to the Bcc to experimentally validate and extend our previous *in silico* analyses (55). Besides the non-Bcc species B. pseudomallei and B. thailandensis, only a few strains of *B. ambifaria* and B. cepacia were already known to carry the *hmqABCDEFG* operon and to produce HMAQs (16, 36, 38–44). Indeed, the presence of the *hmqABCDEFG* operon is well conserved in B. pseudomallei and B. thailandensis, but its prevalence within the Bcc remains unclear (55). To understand the ecological role of the Hmq system, we first needed to evaluate its distribution and prevalence. Since available genomes are not equally distributed between Bcc species, we screened a panel of 312 Bcc strains for the presence of the *hmqABCDEFG* operon by PCR. Since the *hmqG* gene is detectable only when the complete *hmqABCDEFG* operon is present and no significant variation of the *hmqABCDEFG* operon was observed in the 447 positive genomes analyzed by bioinformatics (55), we considered the operon to be present when both *hmqA* and *hmqG* genes were amplified. While the possibility that that some strains carry variations in the *hmqABCDEFG* operon cannot be excluded, we did not detect any in our previous bioinformatic analysis comparing whole genomes (55). The prevalence of the *hmqABCDEFG* operon found here for the Bcc followed the same distribution as that found by *in silico* analysis. Our PCR-based screening could miss some strains carrying the operon due to the limited availability of whole-genome sequences for some Bcc species (e.g., *B. arboris*, *B. metallica*, and *B. stabilis*). Nevertheless, our primers could amplify *hmqA* and *hmqG* targets in species not previously known to carry a *hmqABCDEFG* operon (e.g., *B. vietnamiensis*), which was expected due to the high level of identity (>88%) of *hmqA* and *hmqG* among identified and sequenced Bcc species (55). Furthermore, testing the production of HMAQs in a group of 31 PCR-negative strains confirmed the absence of production of these metabolites. Taking all the results together, we are confident that we identified the strains carrying the genetic ability to synthesize HMAQs.

Based on our previous results obtained with a few *B. ambifaria* isolates, we had hypothesized that the Hmq system would be more prevalent among clinical isolates and produce more HMAQs than environmental ones (36, 43, 46). Unexpectedly, we discovered that B. cenocepacia, *B. multivorans*, and *B. vietnamiensis* (the prominent Bcc species colonizing immunosuppressed individuals and those with CF) transmitted between patients (22, 23, 58) never or rarely (0 of 72 B. cenocepacia, 0 of 35 *B. multivorans*, and 5 of 35 *B. vietnamiensis* strains) carry the *hmqABCDEFG* operon. As this operon in Bcc results from a common ancestor, its absence could result from chromosomic rearrangement (by deletion or replacement) (55). In contrast, our data suggest that the Hmq system could play a beneficial role in niche adaptation to the rhizosphere microbial community due to the large prevalence of the *hmqABCDEFG* operon among environmental strains of *B. ambifaria*, B. cepacia, *B. contaminans*, *B. pyrrocinia*, and *B. ubonensis*, species known for their preference for the plant root environment (19, 59–62). The presence of the Hmq system in clinical strains of these five common environmental species is compatible with a recent or direct environmental acquisition in CF patients (60, 63, 64).

Among the strains carrying the *hmqABCDEFG* operon, not all produced HMAQs under the tested conditions. Since the limit of detection for these molecules was 50 μg/liter, we cannot exclude the possibility that some of these strains actually produced lower levels. For those strains producing HMAQs in TSB, growth in ASM did not inhibit HMAQ production and stimulated a detectable production in 10 additional strains, which could be explained by differences in regulation of the biosynthetic operon. Further, an increase in the number of HMAQ-positive strains when a third culture condition (4 days on TSA plates) was tested highlights the need to use nutritionally appropriate media for investigating the production of secondary metabolites (53). For this purpose, we tried to optimize a medium for HMAQ production. Nevertheless, our results show that the production of HMAQs in the Bcc is dependent on the conditions of growth and that *hmqABCDEFG*-positive strains not producing detectable HMAQs under the conditions tested here could produce enough HMAQs under different ones.

Lack of transcription of the *hmqABCDEFG* operon seemed to explain the observation that 28% of Bcc strains carrying the genes did not produce HMAQs when grown in TSB at 30°C. This result may be observed due to nonpromoter effects (e.g., posttranscriptional or posttranslational control). Indeed, production could be detected simply by changing the culture conditions, suggesting that the promoter of the *hmqABCDEFG* was not expressed, although we cannot exclude the possibility that it could be nonfunctional in some strains. We thus believe the same explanation (poor *hmqABCDEFG* transcription) could apply to the remaining 13% strains for which we could not yet identify appropriate culture conditions. Future work will involve developing a better understanding of the nutritional and regulatory elements (e.g., posttranscriptional or posttranslational regulation) controlling the expression of the *hmqABCDEFG* operon and production of HMAQs.

Because the *hmqABCDEFG* operon is more prevalent in environmental strains, which are often isolated from the rhizosphere, we investigated the impact of the Hmq system on the growth promotion ability of a *B. ambifaria* strain of a model plant, *P. sativum*. The inactivation of *hmqA* or *hmqG* prevented root growth promotion, presumably because of the inability to produce HMAQs. As is the case for cepacin and pyrrolnitrin, HMAQs could influence the rhizosphere microbial community, for instance, due to their antimicrobial activity (16, 36, 39–41, 45, 46, 50, 54, 65) or other properties. As we have previously reported that the HMAQs influence the Cep regulatory system, the molecules could indirectly impact the production of other plant-beneficial metabolites and functions regulated by quorum sensing (e.g., enacyloxin, pyrrolnitrin, cepacin, and antifungal cluster (afc) lipopeptide) (16, 30, 36, 39–41, 45, 46, 50, 54, 65–67). Further experiments with other HMAQ producers and pure HMAQs will provide exciting insights.

## MATERIALS AND METHODS

### Strains and culture conditions.

A total of 312 strains isolated from either clinical or environmental settings were used in this study (Table S1). Uncertain identification was confirmed by amplifying and sequencing the *recA* and *gyrB* genes at the IRCM Sequencing Platform (Montreal) following the directions on the PubMLST database (https://pubmlst.org/organisms/burkholderia-cepacia-complex) (Table S8).

Strains were cultured in borosilicate tubes containing 3 ml tryptic soy broth (TSB; Difco) from stocks frozen at −80°C in 15% glycerol and incubated at 30°C with 60 rpm rotative shaking overnight (∼16 h).

### Detection of the presence of *hmqABCDEFG* operon by PCR.

Genomic DNA was extracted following a previously described method (68). Briefly, cells were resuspended in lysis buffer (50 mM Tris-HCl [pH 8], 5 mM EDTA-2Na [pH 8], 3% SDS) and transferred to a tube containing glass beads. The cells were lysed in a Fast-Prep-24 instrument (MP Biomedicals). Then the lysates were centrifuged at 8,000 × *g* for 5 min, the supernatants were transferred to a new tube, and 2.5 N ammonium acetate was added. The supernatant was transferred to a new tube, and 1 volume of isopropanol was added. The pellets were washed with 75% ethanol and dried before resuspension in 50 μl water.

For the strains acquired from the Burkholderia cepacia Research Laboratory and Repository and from the Canadian Burkholderia cepacia complex Research and Referral Repository, genomic DNA was extracted using a 96-well plate gDNA extraction kit (Favorgen, Canada).

The *hmqA* and *hmqG* genes were amplified by PCR using EasyTaq polymerase (Transgen, Canada). Primers were designed based on a consensus sequence of 11 complete Bcc sequences available in 2017 (Table S8). A strain was considered to have a complete *hmqABCDEFG* operon when amplification of both *hmqA* and *hmqG* targets was attained, based on our previous analyses (55).

### Detection of the presence of the pc3 chromosome and the *hmqABCDEFG* operon in Bcc strains.

Assembled genomes are available on DB Burkholderia (http://www.Burkholderia.com). We selected only complete assembled genomes and searched for the presence of the *hmqABCDEFG* operon by orthology and homology directly on DB Burkholderia. Results are listed in Table S3.

### Phylogeny of the Bcc based on MLST and *hmqABCDEFG* sequences.

First, *atpD*, *gltB*, *gyrB*, *recA*, *lepA*, *phaC*, and *trpB* gene sequences were used to generate a Bcc phylogeny based on MLST. Second, the *hmqABCDEFG* operon sequence was used to determine the coevolution or horizontal transfer of the Hmq system within Bcc species. Sequences were found on DB Burkholderia (http://www.Burkholderia.com) and concatenated in the order *atpD*, *gyrB*, *recA*, *gltB*, *lepA*, *phaC*, and *trpB*, with *hmqABCDEFG* in parallel. The resulting concatenated sequences were aligned using Clustal Omega (69), and trees were generated by RAxML using the GTRGAMMA model and 1,000 bootstraps. The branches are labeled where bootstrap values are >50.

### Quantification of HMAQ production by LC-MS/MS.

Bcc strains were cultured in 5 ml TSB at a starting optical density at 600 nm (OD_600_) of 0.05 and incubated at 30°C with shaking for an overnight. 5,6,7,8-Tetradeutero-4-hydroxy-2-heptylquinoline (HHQ-d4) was used as an internal standard (70). The total HMAQs were extracted from 4 ml culture with 1 volume of ethyl acetate. After nitrogen evaporation, the residues were dissolved in 400 μl high-performance liquid chromatography (HPLC)-grade acetonitrile. Samples were analyzed by liquid chromatography coupled with mass spectrometry (LC-MS) in positive electrospray ionization using a Kinetex 5-μm EVO C_18_ 100-Å 100- by 3-mm reverse-phase column as previously described (70). A Quattro Premier XE triple quadrupole was used as a detector (Waters). A multiple reaction monitoring (MRM) program was used to detect HMAQ families (36). This experiment was conducted with three independent biological replicates.

### ASM, TSB, and TSA medium assays.

The strains were grown in ASM liquid medium (71) and TSA agar plates out from overnight cultures and incubated at 30°C for 24 h and 4 days, respectively, or in grown TSB and incubated at 37°C for 24 h.

One milliliter of ASM culture was extracted as described for the HMAQ production method. For each TSA plate, 5 ml water was added to extract the HMAQs from the agar. For each sample, 1 ml was extracted with 1 volume ethyl acetate containing 4 ppm HHQ-D4, concentrated 10 times, dissolved in HPLC-grade acetonitrile, and analyzed as described above for the HMAQ extraction method.

The experiments were performed in two independent biological replicates.

### Detection of the expression of the *hmqABCDEFG* operon by RT-PCR.

Total RNA was extracted from cultures grown in TSB to an OD_600_ of 3.0 using TransZol (Transgene, Canada) by following the manufacturer’s instructions. Residual DNA was removed using the Turbo DNase (Thermo Fisher, Canada). Reverse transcription was performed using the iScript kit (Bio-Rad, Canada). The expression of the *hmqABCDEFG* operon was determined by PCR targeting *hmqA.* The *ndh* gene served as a reference gene (Table S8) (72).

### Correlation between the presence of the *hmqABCDEFG* operon and the production of HMAQs in Bcc with different characteristics.

Based on our qualitative data (Table 2 and Table S7), we studied the correlation by Fisher’s exact test for count data using R software (http://www.R-project.org) (73).

### Pisum sativum growth promotion.

Pisum sativum seeds were decontaminated using successive bleach and 70% ethanol treatments (10 min each, under shaking [60 rpm], 3 times) and then germinated for 4 days at room temperature on 0.8% agar plates. *B. ambifaria* HSJ1 and isogenic *hmqA*::pKnock-Cm, and *hmqG*::pKnock-Cm mutants, previously respectively generated by integrating a 757-bp internal fragment of *hmqA* and a 525-bp fragment of *hmqG* from HSJ1 into the suicide vector pKnock-Cm and by selecting single-crossover insertion mutants obtained following mating Escherichia coli SM10 (pKnock-Cm-*hmqA* or pKnock-Cm-*hmqG*) with *B. ambifaria* HSJ1 (36), were incubated at 30°C overnight in TSB with shaking at 60 rpm. Seeds were exposed by adding 10^5^ bacteria/ml in 5 ml Murashige and Skoog (MS) basal medium (Sigma) (43). Plants were grown at room temperature for 5 days. Roots were then cut, dried at 52°C overnight, and weighed. A Dunn test was used for statistical analyses. This experiment was repeated twice, with the same conclusions.
