# Supplementary material for: Presence of the Hmq System and Production of 4-Hydroxy-3-Methyl-2-Alkylquinolines Are Heterogeneously Distributed between Burkholderia cepacia Complex Species and More Prevalent among Environmental than Clinical Isolates
Source: Microbiol Spectr. 2021 Jun 16;9(1):10.1128/spectrum.00127-21. doi: 10.1128/spectrum.00127-21 (PMC8552760; doi:10.1128/spectrum.00127-21)
Supplement: SUPPLEMENTAL FILE 1 — Supplemental material. Download SPECTRUM00127-21_Supp_1_seq10.docx, DOCX file, 1.1 MB [file spectrum00127-21_supp_1_seq10.docx]

**4Supplemental Material**

**Presence of the Hmq system and production of 4-hydroxy-3-methyl-2-alkylquinolines is heterogeneously distributed between *Burkholderia cepacia* complex species and more prevalent among environmental than clinical isolates**

Running title: The Hmq system is more frequent among environmental Bcc

Pauline M.L. Coulon^A^, James E.A. Zlosnik^B^, & Eric Déziel^A#^

^A^Centre Armand-Frappier Santé Biotechnologie, Institut National de la Recherche Scientifique (INRS), Laval, QC, Canada

^B^Canadian *Burkholderia cepacia* Complex Research and Referral Repository, University of British Columbia, Vancouver, BC, Canada

Corresponding author: Eric Déziel, [eric.deziel@inrs.ca](mailto:eric.deziel@inrs.ca)

Consensus TGCTTTCCGSAGCAYGTGCTCMRGCCYACSGCCGAHGACGTSTTCTGCGGRTCGCCR**CYG** 720

B_ubonensis_MSMB22 .........C....C......AA...C..G.....C.....C........A.....G**.C.** 720

B_stagnalis_MSMB777WGS .........C....T......AA...C..G.....T.....C........A.....G**.C.** 720

B_stagnalis_MSMB1956WGS .........C....C......AA...C..G.....T.....C........A.....G**.C**. 720

B_territorii_MSMB1502WGS .........C....C......CG...C..G.....A.....C........A.....G.T. 720

B_territorii_MSMB1301 .........C....C......CG...C..G.....A.....C........A.....G.C. 720

B_cepacia_ATCC25416 .........C....C......CG...C..G.....C.....C........A.....G.C. 720

B_lata_LK13 .........C....C......CG...C..G.....T.....C........A.....G.C. 720

B_ubonensis_MSMB867 .........C....C......AA...C..G.....C.....C........A.....G.C. 720

B_stabilis_EB159 .........C....C......CG...C..G.....C.....C........G.....A.C. 720

B_stabilis_LA20W .........C....C......AG...C..G.....T.....C........G.....A.C. 720

B_pyrrocinia_CH-67 .........C....C......AA...C..G.....T.....C........A.....G.C. 720

B_contaminans_FFH2055 .........C....C......AA...C..G.....T.....C........G.....G.C. 720

B_contaminans_MS14 .........C....C......AA...T..G.....T.....C........G.....G.C. 720

B_ambifaria_AMMD .........G....C......CG...C..C.....T.....G........A.....G.C. 720

B_ambifaria_CEP0996 .........G....C......CG...C..C.....T.....G........A.....G.C. 720

B_ambifaria_HSJ1 .........G....C......CG...C..C.....T.....G........A.....G.C. 720

B_cepacia_LK29 .........C....C......AG...C..G.....T.....C........G.....A.C. 720

B_pyrrocinia_DSM10685 .........C....C......AG...C..G.....T.....C........G.....A.C. 720

Consensus **CTCGCGTTYACGTTYGG**SCTCGGCGCRCTGYTGCTGTTTCCRRTCAGYGTCGGCGCRAGC 780

B_ubonensis_MSMB22 ........C.....T..C........G...C..........GG....C........G... 780

B_stagnalis_MSMB777WGS ........C.....C..C........G...C..........GG....C........G... 780

B_stagnalis_MSMB1956WGS ........C.....C..C........G...C..........GG....C........G... 780

B_territorii_MSMB1502WGS ........C.....C..C........G...C..........GG....C........G... 780

B_territorii_MSMB1301 ........C.....C..C........G...C..........GG....C........G... 780

B_cepacia_ATCC25416 ........C.....C..C........G...C..........AG....C........G... 780

B_lata_LK13 ........C.....C..C........G...C..........GG....C........A... 780

B_ubonensis_MSMB867 ........C.....T..C........G...C..........GG....C........G... 780

B_stabilis_EB159 ........C.....C..C........A...C..........GA....T........A... 780

B_stabilis_LA20W ........C.....C..C........G...C..........GA....C........G... 780

B_pyrrocinia_CH-67 ........C.....C..C........G...C..........GG....C........G... 780

B_contaminans_FFH2055 ........T.....C..C........G...C..........GA....T........G... 780

B_contaminans_MS14 ........T.....C..C........G...C..........GA....T........G... 780

B_ambifaria_AMMD ........C.....C..G........G...C..........GA....T........G... 780

B_ambifaria_CEP0996 ........C.....C..G........G...C..........GA....T........G... 780

B_ambifaria_HSJ1 ........C.....C..G........G...C..........GA....T........G... 780

B_cepacia_LK29 ........C.....C..C........G...C..........GA....T........A... 780

B_pyrrocinia_DSM10685 ........C.....C..C........G...T..........GA....C........G... 780

Consensus GACRCGCGRCAACGYGACTACGTSARGYA**CGGYTGGAACCTGACGGG**SGAYRSCGCSTAY 1260

B_ubonensis_MSMB22 ...G....G.....C........C.G.C....C..............G..TGC...C..C 1251

B_stagnalis_MSMB777WGS ...G....G.....C........G.A.C....C..............C..CAG...C..C 1251

B_stagnalis_MSMB1956WGS ...G....G.....C........G.A.C....C..............C..CAG...C..C 1251

B_territorii_MSMB1502WGS ...A....G.....C........C.A.C....T..............C..CAG...C..C 1260

B_territorii_MSMB1301 ...A....G.....C........C.A.C....T..............C..CAG...C..C 1260

B_cepacia_ATCC25416 ...A....G.....C........C.A.C....C..............C..CAG...C..C 1260

B_lata_LK13 ...A....A.....C........C.A.C....C..............C..CAG...C..C 1260

B_ubonensis_MSMB867 ...G....G.....C........C.G.C....C..............C..TGC...C..T 1251

B_stabilis_EB159 ...G....G.....C........G.A.C....C..............C..CAG...C..C 1251

B_stabilis_LA20W. ...G....G.....C........G.A.C....C..............C..CAG...C..C 1251

B_pyrrocinia_CH-67 ...G....G.....T........G.A.C....C..............C..CAG...G..C 1251

B_contaminans_FFH2055 ...G....G.....C........C.A.C....C..............C..CAG...G..C 1251

B_contaminans_MS14 ...G....G.....C........C.A.C....C..............C..CAG...G..C 1251

B_ambifaria_AMMD ...A....G.....C........C.A.T....C..............C..CAG...C..C 1251

B_ambifaria_CEP0996 ...A....G.....C........C.A.T....C..............C..CAG...C..C 1251

B_ambifaria_HSJ1 ...A....G.....C........C.A.T....C..............C..CAG...C..C 1251

B_cepacia_LK29 ...G....G.....C........C.A.C....C..............C..CAG...C..C 1251

B_pyrrocinia_DSM10685 ...G....G.....C........G.A.C....C..............C..CAG...C..C 1251

Consensus TGCGTCGAGCGGCGCCGGVTCBGGGCC**SGCGTCGCASGAAATCACG**ACVAARATCCTGCR 10766

B_ubonensis_MSMB22 ..................C..G.....G........G...........G..A.......G 10716

B_stagnalis_MSMB777WGS ..................G..G.....G........G...........G..G.......G 10620

B_stagnalis_MSMB1956WGS ..................G..G.....C........G...........G..G.......G 10608

B_territorii_MSMB1502WGS ..................G..T.....G........G...........G..G.......G 10630

B_territorii_MSMB1301 ..................G..T.....G........G...........G..G.......G 10640

B_cepacia_ATCC25416 ..................A..G.....G........G...........G..G.......G 10649

B_lata_LK13 ..................G..G.....G........G...........G..G.......G 10667

B_ubonensis_MSMB867 ..................G..G.....G........G...........C..A.......G 10653

B_stabilis_EB159 ..................G..C.....G........G...........G..A.......G 10624

B_stabilis_LA20W ..................G..C.....G........G...........G..A.......A 10621

B_pyrrocinia_CH-67 ..................G..G.....G........G...........G..G.......G 10637

B_contaminans_FFH2055 ..................G..C.....G........G...........G..A.......G 10614

B_contaminans_MS14 ..................G..C.....G........G...........G..A.......G 10611

B_ambifaria_AMMD ..................G..C.....G........G...........A..A.......G 10637

B_ambifaria_CEP0996 ..................G..C.....G........G...........A..A.......G 10637

B_ambifaria_HSJ1 ..................G..C.....G........G...........A..A.......G 10637

B_cepacia_LK29 ..................G..C.....C........C...........G..A.......G 10628

B_pyrrocinia_DSM10685 ..................G..C.....G........G...........G..A.......A 10628

Consensus VCCSSAVRSGTGGYTGCGCACCGARCAGCTKCCGTCGGTRTTCAAYTGG**GGCATGCAYTT** 11546

B_ubonensis_MSMB22 A..GG.AGG....T..........G.....G........G.....T...........T.. 11496

B_stagnalis_MSMB777WGS G..CG.CGC....C..........G.....T........G.....C...........C.. 11400

B_stagnalis_MSMB1956WGS G..CC.CGC....C..........G.....T........G.....C...........C.. 11388

B_territorii_MSMB1502WGS G..CG.CGC....C..........G.....G........G.....C...........C.. 11410

B_territorii_MSMB1301 G..CG.CGC....C..........G.....G........G.....C...........C.. 11420

B_cepacia_ATCC25416 C..GG.CAC....T..........G.....T........G.....C...........T.. 11429

B_lata_LK13 C..GG.CAC....T..........G.....T........G.....C...........T.. 11447

B_ubonensis_MSMB867 A..GG.CGC....T..........G.....G........G.....C...........T.. 11433

B_stabilis_EB159 G..GG.CAC....T..........G.....T........G.....C...........T.. 11404

B_stabilis_LA20W G..GG.CAC....T..........G.....T........G.....C...........T.. 11401

B_pyrrocinia_CH-67 G..CG.GGC....C..........G.....T........G.....C...........C.. 11417

B_contaminans_FFH2055 G..GG.CAC....T..........A.....T........G.....C...........T.. 11394

B_contaminans_MS14 G..GG.CAC....T..........G.....T........G.....C...........T.. 11391

B_ambifaria_AMMD G..GG.CAC....T..........G.....T........A.....C...........T.. 11417

B_ambifaria_CEP0996 G..GG.CAC....T..........G.....T........A.....C...........T.. 11417

B_ambifaria_HSJ1 G..GG.CAC....T..........G.....T........A.....C...........T.. 11417

B_cepacia_LK29 G..GG.CAC....T..........G.....T........G.....C...........T.. 11408

B_pyrrocinia_DSM10685 G..GG.CAC....T..........G.....T........G.....C...........T.. 11408

Consensus **YGTGTCGCG**BCARTTCATGGCGCCGTTCAACGARTTCAYRCTYGCRYTGACSCTSGCYGG 11606

B_ubonensis_MSMB22 C........C..G....................A....TG..C..GT....G..G..C.. 11556

B_stagnalis_MSMB777WGS C........G..G....................A....TG..C..GT....G..G..C.. 11460

B_stagnalis_MSMB1956WGS C........G..G....................A....TG..C..GT....G..G..C.. 11448

B_territorii_MSMB1502WGS T........T..G....................G....CA..T..AC....G..G..C.. 11470

B_territorii_MSMB1301 T........T..G....................G....CA..T..AC....G..G..C.. 11480

B_cepacia_ATCC25416 C........C..G....................A....CG..C..GC....C..G..C.. 11489

B_lata_LK13 C........C..G....................A....CG..C..GC....C..G..C.. 11507

B_ubonensis_MSMB867 C........C..G....................A....TG..C..GT....G..G..C.. 11493

B_stabilis_EB159 C........T..G....................A....CG..C..GT....G..G..C.. 11464

B_stabilis_LA20W C........T..G....................A....CG..C..GT....C..C..C.. 11461

B_pyrrocinia_CH-67 C........C..G....................A....CG..C..GC....C..G..C.. 11477

B_contaminans_FFH2055 C........T..A....................A....CG..T..GT....C..G..C.. 11454

B_contaminans_MS14 C........T..A....................A....CG..T..GT....C..G..C.. 11451

B_ambifaria_AMMD C........C..A....................A....CA..C..GT....C..G..T.. 11477

B_ambifaria_CEP0996 C........C..A....................A....CA..C..GT....C..G..T.. 11477

B_ambifaria_HSJ1 C........C..A....................A....CA..C..GT....C..G..T.. 11477

B_cepacia_LK29 C........T..G....................A....CG..C..GT....G..G..C.. 11468

B_pyrrocinia_DSM10685 C........T..G....................A....CG..C..GT....C..C..C.. 11468

**Figure S1. Partial alignment of hmqA and hmqG genes and primers’ binding sequences.**

Sequences were aligned using Clustal Omega (1). Consensus sequences corresponding to hmqA primers are in red and in green for hmqG primers. These primers were used for the screening.

**Figure S2. Presence of the *hmqABCDEFG* operon in the genomes of Bcc strains**

The results are in percentages of total strains for each origin. Numbers represent the number of strains in each species and for each origin, carrying (white) or not (black) the *hmqABCDEFG* operon.

**Figure S3. Distribution of HMAQ production among Bcc species**

HMAQs have been quantified by LC/MS with a limit of detection of 50 µg/L for each molecule in the total culture. Results are presented as percentages of total strains for each origin. Numbers represent the number of strains in each species and for each origin, producing (white) or not (black) HMAQs.

**Figure S4. Concentration of HMAQs and HHQ produced by Bcc species when cultured in TSB.**

HMAQs and HHQ have been quantified by LC/MS with a limit of detection of 50 µg/L for each molecule in the total culture. Tested strains have been grouped by species. The production of HMAQs and HHQ have been quantified in three biological replicates for each strain. Red lines and numbers represent the average of production with the clinical and environmental strains.

**** **Figure S5. Expression of the hmqA gene in strains the main species of Bcc having the hmqABCDEFG operon but for which HMAQ production was not detected.** A) RT-PCR on B. cepacia and B. contaminans strains. B) RT-PCR on B. contaminans and B. ambifaria strains. C) RT-PCR on B. vietnamiensis strains. Black and white arrows indicate ndh and hmqA amplicons respectively.

**Table S1. List of strains investigated in this study**

| **Strains** | | **Type** | **References** | **Other names** |
| --- | --- | --- | --- | --- |
| ***Burkholderia ambifaria*** | | |  | |
|  | *B. ambifaria* AMMD | Soil, rhizosphere (USA) | (2) | LMG19182/FC0768/BCC0588 |
|  | *B. ambifaria* AU0212 | CF isolate (USA) | (3) |  |
|  | *B. ambifaria* AU4157 | Clinical isolate | BcRLR |  |
|  | *B. ambifaria* AU7994 | Clinical isolate |  |  |
|  | *B. ambifaria* AU8235 | Clinical isolate | BcRLR |  |
|  | *B. ambifaria* CEP0516 | CF isolate (Australia) | (2) |  |
|  | *B. ambifaria* CEP0617 | Clinical isolate | (2) | LMG-P 24636 |
|  | *B. ambifaria* CEP0958 | CF isolate (Australia) | (2) |  |
|  | *B. ambifaria* CEP0990 | Clinical isolate |  |  |
|  | *B. ambifaria* CEP0996 | CF isolate (Australia) | (4) | LMG 19467 |
|  | *B. ambifaria* CEP1231 | Clinical isolate |  |  |
|  | *B. ambifaria* ES0020 | Environmental isolate, Oregon (USA) | BcRLR |  |
|  | *B. ambifaria* HI2425 | Soil, New York (USA) | BcRLR |  |
|  | *B. ambifaria* HI2468 | Pea rhizosphere, Wisconsin (USA) | BcRLR |  |
|  | *B. ambifaria* HI2482 | Soil, New York (USA) | BcRLR |  |
|  | *B. ambifaria* HI2626 | Soil, rice field, New York (USA) | BcRLR |  |
|  | *B. ambifaria* HI2672 | Soil, rice field, New York (USA) | BcRLR |  |
|  | *B. ambifaria* HI3544 | Soil, North Carolina (USA) | BcRLR |  |
|  | *B. ambifaria* HI3590 | Environmental isolate (USA) | BcRLR |  |
|  | *B. ambifaria* HI3687 | Soil, North Carolina (USA) | BcRLR |  |
|  | *B. ambifaria* HI3709 | Soil, North Carolina (USA) | BcRLR |  |
|  | *B. ambifaria* HI3738 | Soil, Michigan (USA) | BcRLR |  |
|  | *B. ambifaria* HI3890 | Soil, Illinois (USA) | BcRLR |  |
|  | *B. ambifaria* HSJ1 | CF isolate (Canada) | (5) |  |
|  | *B. ambifaria* HSJ1 pKnock::*hmqA* Cm |  |  |  |
|  | *B. ambifaria* HSJ1 pKnock::*hmqG* Cm |  |  |  |
|  | *B. ambifaria* IOP40-10 | Environmental isolate | J. Tiedje |  |
|  | *B. ambifaria* LMG17828 |  | (2) | ATCC 53266/FC0662 |
|  | *B. ambifaria* MW2073 | Environmental isolate |  |  |
|  | *B. ambifaria* PC736 | Environmental isolate (USA) | BcRLR |  |
|  | *B. ambifaria* PHP7 | Environmental isolate | (2) |  |
|  | *B. ambifaria* VC11631 | CF isolate, Quebec (Canada) | CBCCRRR |  |
|  | *B. ambifaria* VC15422 | CF isolate, Ontario (Canada) | CBCCRRR |  |
|  | *B. ambifaria* VC16196 | Clinical isolate, Quebec (Canada) | CBCCRRR |  |
| ***Burkholderia anthina*** | | |  | |
|  | *B. anthina* HI3538 | Soil, North Carolina (USA) | BcRLR | Bcc indeterminate 7 HI3538 |
|  | *B. anthina* HI3655 | Soil, North Carolina (USA) | BcRLR |  |
|  | *B. anthina* VC15382 | Clinical isolate, Quebec (Canada) | CBCCRRR |  |
|  | *B. anthina* VC16083 | Clinical isolate, Quebec (Canada) | CBCCRRR |  |
| ***Burkholderia arboris*** | | |  | |
|  | *B. arboris* ES0222 | Environmental isolate, Pennsylvania (USA) | BcRLR |  |
|  | *B. arboris* ES0263 | Soil, Pennsylvania (USA) | BcRLR |  |
|  | *B. arboris* VC10224 | Clinical isolate (Canada) | CBCCRRR |  |
|  | *B. arboris* VC8833 | CF isolate, New Brunswick (Canada) | CBCCRRR |  |
| ***Burkholderia cenocepacia*** | | |  | |
|  | *B.* *cenocepacia* CEP024 | CF isolate isolate (Canada) | Speert collection |  |
|  | *B.* *cenocepacia* CEP0511 | CF isolate isolate (Australia) | (6) | LMG 18830 |
|  | *B.* *cenocepacia* CEP0565 | Clinical isolate |  |  |
|  | *B. cenocepacia* ES1405 | Environmental isolate, Ontario (Canada) | BcRLR |  |
|  | *B. cenocepacia* HI2424 | Soil, New York (USA) | BcRLR |  |
|  | *B. cenocepacia* HI2606 | Soil, New York (USA) | BcRLR |  |
|  | *B. cenocepacia* HI2876 | Dialysis water, Missouri (USA) | BcRLR |  |
|  | *B. cenocepacia* HI2976 | Sink, North Carolina (USA) | BcRLR |  |
|  | *B. cenocepacia* HI3540 | Soil, North Carolina (USA) | BcRLR |  |
|  | *B. cenocepacia* HI3855 | Contaminated mouthwash, South Carolina (USA) | BcRLR |  |
|  | *B. cenocepacia* HI4004 | Plumbing biofilm, Washington (USA) | BcRLR |  |
|  | *B. cenocepacia* HI4101 | Doctor examination room, Illinois (USA) | BcRLR |  |
|  | *B. cenocepacia* HI4143 | Temperature probe, Arizona (USA) | BcRLR |  |
|  | *B. cenocepacia* HI4261 | Chlorhexidine body wipe, Kansas (USA) | BcRLR |  |
|  | *B. cenocepacia* HI4437 | Contaminated lotion, Massachusetts (USA) | BcRLR |  |
|  | *B. cenocepacia* HI4904 | Contaminated cleansing foam, Pennsylvainia (USA) | BcRLR |  |
|  | *B.* *cenocepacia* K56-2 | CF isolate isolate (Canada) | (7) |  |
|  | *B.* *cenocepacia* IIIA VC10277 | CF isolate, Alberta (Canada) | CBCCRRR |  |
|  | *B.* *cenocepacia* IIIA VC12308 | CF isolate, British Columbia (Canada) | CBCCRRR |  |
|  | *B.* *cenocepacia* IIIA VC13139 | CF isolate, Quebec (Canada) | CBCCRRR |  |
|  | *B.* *cenocepacia* IIIA VC14610 | CF isolate, New Brunswick (Canada) | CBCCRRR |  |
|  | *B.* *cenocepacia* IIIA VC15419 | CF isolate, British Columbia (Canada) | CBCCRRR |  |
|  | *B.* *cenocepacia* IIIA VC15451 | CF isolate, Ontario (Canada) | CBCCRRR |  |
|  | *B.* *cenocepacia* IIIA VC16156 | CF isolate, British Columbia (Canada) | CBCCRRR |  |
|  | *B.* *cenocepacia* IIIA VC16199 | CF isolate, Alberta (Canada) | CBCCRRR |  |
|  | *B.* *cenocepacia* IIIA VC16873 | CF isolate, New Brunswick (Canada) | CBCCRRR |  |
|  | *B.* *cenocepacia* IIIA VC16874 | CF isolate, New Brunswick (Canada) | CBCCRRR |  |
|  | *B.* *cenocepacia* IIIA VC17671 | CF isolate, Quebec (Canada) | CBCCRRR |  |
|  | *B.* *cenocepacia* IIIA VC17819 | CF isolate, Alberta (Canada) | CBCCRRR |  |
|  | *B.* *cenocepacia* IIIA VC18585 | CF isolate, British Columbia (Canada) | CBCCRRR |  |
|  | *B.* *cenocepacia* IIIA VC18609 | CF isolate, Quebec (Canada) | CBCCRRR |  |
|  | *B.* *cenocepacia* IIIA VC18996 | CF isolate, New Brunswick (Canada) | CBCCRRR |  |
|  | *B.* *cenocepacia* IIIA VC18999 | CF isolate, New Brunswick (Canada) | CBCCRRR |  |
|  | *B.* *cenocepacia* IIIA VC3917 | CF isolate, New Brunswick (Canada) | CBCCRRR |  |
|  | *B.* *cenocepacia* IIIA VC5069 | CF isolate, Quebec (Canada) | CBCCRRR |  |
|  | *B.* *cenocepacia* IIIA VC5621 | CF isolate, Ontario (Canada) | CBCCRRR |  |
|  | *B.* *cenocepacia* IIIA VC6356 | CF isolate, British Columbia (Canada) | CBCCRRR |  |
|  | *B.* *cenocepacia* IIIA VC6553 | CF isolate, British Columbia (Canada) | CBCCRRR |  |
|  | *B.* *cenocepacia* IIIA VC8286 | CF isolate, Alberta (Canada) | CBCCRRR |  |
|  | *B.* *cenocepacia* IIIA VC8356 | CF isolate, Quebec (Canada) | CBCCRRR |  |
|  | *B.* *cenocepacia* IIIA VC8426 | CF isolate, British Columbia (Canada) | CBCCRRR |  |
|  | *B.* *cenocepacia* IIIA VC8607 | CF isolate, Alberta (Canada) | CBCCRRR |  |
|  | *B.* *cenocepacia* IIIA VC8611 | CF isolate, Alberta (Canada) | CBCCRRR |  |
|  | *B.* *cenocepacia* IIIA VC8614 | CF isolate, Alberta (Canada) | CBCCRRR |  |
|  | *B.* *cenocepacia* IIIA VC9080 | CF isolate, British Columbia (Canada) | CBCCRRR |  |
|  | *B.* *cenocepacia* IIIA VC9296 | CF isolate, Newfoundland and Labrador (Canada) | CBCCRRR |  |
|  | *B.* *cenocepacia* IIIB VC11311 | CF isolate, British Columbia (Canada) | CBCCRRR |  |
|  | *B.* *cenocepacia* IIIB VC11653 | CF isolate, Quebec (Canada) | CBCCRRR |  |
|  | *B.* *cenocepacia* IIIB VC13104 | CF isolate, Ontario (Canada) | CBCCRRR |  |
|  | *B.* *cenocepacia* IIIB VC13187 | CF isolate, Ontario (Canada) | CBCCRRR |  |
|  | *B.* *cenocepacia* IIIB VC14376 | CF isolate, Ontario (Canada) | CBCCRRR |  |
|  | *B.* *cenocepacia* IIIB VC14524 | CF isolate, Alberta (Canada) | CBCCRRR |  |
|  | *B.* *cenocepacia* IIIB VC14529 | CF isolate, Quebec (Canada) | CBCCRRR |  |
|  | *B.* *cenocepacia* IIIB VC15122 | CF isolate, British Columbia (Canada) | CBCCRRR |  |
|  | *B.* *cenocepacia* IIIB VC15240 | CF isolate, Ontario (Canada) | CBCCRRR |  |
|  | *B.* *cenocepacia* IIIB VC15241 | CF isolate, Ontario (Canada) | CBCCRRR |  |
|  | *B.* *cenocepacia* IIIB VC16932 | CF isolate, Nova Scotia (Canada) | CBCCRRR |  |
|  | *B.* *cenocepacia* IIIB VC17657 | CF isolate, Quebec (Canada) | CBCCRRR |  |
|  | *B.* *cenocepacia* IIIB VC18097 | CF isolate, Quebec (Canada) | CBCCRRR |  |
|  | *B.* *cenocepacia* IIIB VC18107 | CF isolate, British Columbia (Canada) | CBCCRRR |  |
|  | *B.* *cenocepacia* IIIB VC18236 | CF isolate, British Columbia (Canada) | CBCCRRR |  |
|  | *B.* *cenocepacia* IIIB VC18454 | CF isolate, Quebec (Canada) | CBCCRRR |  |
|  | *B.* *cenocepacia* IIIB VC18569 | CF isolate, Quebec (Canada) | CBCCRRR |  |
|  | *B.* *cenocepacia* IIIB VC18658 | CF isolate, British Columbia (Canada) | CBCCRRR |  |
|  | *B.* *cenocepacia* IIIB VC5625 | CF isolate, Ontario (Canada) | CBCCRRR |  |
|  | *B.* *cenocepacia* IIIB VC6598 | CF isolate, British Columbia (Canada) | CBCCRRR |  |
|  | *B.* *cenocepacia* IIIB VC7349 | CF isolate, British Columbia (Canada) | CBCCRRR |  |
|  | *B.* *cenocepacia* IIIB VC7849 | CF isolate, British Columbia (Canada) | CBCCRRR |  |
|  | *B.* *cenocepacia* IIIB VC7911 | CF isolate, British Columbia (Canada) | CBCCRRR |  |
|  | *B.* *cenocepacia* IIIB VC8340 | CF isolate, British Columbia (Canada) | CBCCRRR |  |
|  | *B.* *cenocepacia* IIIB VC8870 | CF isolate, Manitoba (Canada) | CBCCRRR |  |
|  | *B.* *cenocepacia* IIIB VC9859 | CGD, Ontario (Canada) | CBCCRRR |  |
| ***Burkholderia cepacia*** | | |  | |
|  | *B. cepacia* ATCC25416 | Onion root | (8) | LMG1222/CEP0031 |
|  | *B. cepacia* CEP0509 | CF isolate (Australia) | (9) | LMG18821 |
|  | *B. cepacia* BTS13 | CF isolate (Italy) | (10) |  |
|  | *B. cepacia* HI2430 | Soil, New York (USA) | BcRLR |  |
|  | *B. cepacia* HI2563 | Soil, New York (USA) | BcRLR |  |
|  | *B. cepacia* HI2578 | Soil, New York (USA) | BcRLR |  |
|  | *B. cepacia* HI2615 | Soil, celery field, New York (USA) | BcRLR |  |
|  | *B. cepacia* HI2671 | Soil, rice field, New York (USA) | BcRLR |  |
|  | *B. cepacia* HI2741 | Contaminated mouthwash, New York (USA) | BcRLR |  |
|  | *B. cepacia* HI3312 | Dialysis machine, Florida (USA) | BcRLR |  |
|  | *B. cepacia* HI3551 | Soil, North Carolina (USA) | BcRLR |  |
|  | *B. cepacia* HI3708 | Soil, North Carolina (USA) | BcRLR |  |
|  | *B. cepacia* HI3895 | Soil, Illinois (USA) | BcRLR |  |
|  | *B. cepacia* HI4577 | Hospital sink drain (USA) | BcRLR |  |
|  | *B. cepacia* VC13132 | CF isolate, Ontario (Canada) | CBCCRRR |  |
|  | *B. cepacia* VC13196 | CF isolate, Alberta (Canada) | CBCCRRR |  |
|  | *B. cepacia* VC13394 | CF isolate, Alberta (Canada) | CBCCRRR |  |
|  | *B. cepacia* VC13575 | CF isolate, Ontario (Canada) | CBCCRRR |  |
|  | *B. cepacia* VC14106 | CF isolate, British Columbia (Canada) | CBCCRRR |  |
|  | *B. cepacia* VC14457 | CF isolate, British Columbia (Canada) | CBCCRRR |  |
|  | *B. cepacia* VC16383 | Clinical isolate, Quebec (Canada) | CBCCRRR |  |
|  | *B. cepacia* VC16708 | CF isolate, Quebec (Canada) | CBCCRRR |  |
|  | *B. cepacia* VC17333 | CF isolate, Alberta (Canada) | CBCCRRR |  |
|  | *B. cepacia* VC17746 | Non-CF isolate, Quebec (Canada) | CBCCRRR |  |
|  | *B. cepacia* VC17928 | CF isolate, Quebec (Canada) | CBCCRRR |  |
|  | *B. cepacia* VC18315 | CF isolate, British Columbia (Canada) | CBCCRRR |  |
|  | *B. cepacia* VC18839 | Non-CF isolate, Quebec (Canada) | CBCCRRR |  |
|  | *B. cepacia* VC18842 | Non-CF isolate, Quebec (Canada) | CBCCRRR |  |
|  | *B. cepacia* VC19225 | CF isolate, British Columbia (Canada) | CBCCRRR |  |
|  | *B. cepacia* VC19276 | CF isolate, Quebec (Canada) | CBCCRRR |  |
|  | *B. cepacia* VC9490 | CF isolate, British Columbia (Canada) | CBCCRRR |  |
| ***Burkholderia contaminans*** | | |  | |
|  | *B. contaminans* FFH2055 | CF isolate (Argentina) | (11) |  |
|  | *B. contaminans* HI3422 | Contaminated nasal spray, Colorado (USA) | BcRLR |  |
|  | *B. contaminans* HI3570 | Soil, North Carolina (USA) | BcRLR |  |
|  | *B. contaminans* HI3852 | Trypan blue opthalmic solution (USA) | BcRLR |  |
|  | *B. contaminans* HI3887 | Pharmacy IV fluid, Missouri (USA) | BcRLR |  |
|  | *B. contaminans* HI4067 | Environmental isolate (Argentina) | BcRLR |  |
|  | *B. contaminans* HI4232 | Contaminated mouthwash, Arizona (USA) | BcRLR |  |
|  | *B. contaminans* HI4402 | Water, Kentuky(USA) | BcRLR |  |
|  | *B. contaminans* VC14347 | CF isolate, British Columbia (Canada) | CBCCRRR |  |
|  | *B. contaminans* VC15406 | CF isolate, British Columbia (Canada) | CBCCRRR |  |
|  | *B. contaminans* VC16087 | CF isolate, British Columbia (Canada) | CBCCRRR |  |
|  | *B. contaminans* VC16848-b | CF isolate, British Columbia (Canada) | CBCCRRR |  |
|  | *B. contaminans* VC16897 | Clinical isolate, Quebec (Canada) | CBCCRRR |  |
|  | *B. contaminans* VC16948 | CF isolate, British Columbia (Canada) | CBCCRRR |  |
|  | *B. contaminans* VC19056 | CF isolate, British Columbia (Canada) | CBCCRRR |  |
|  | *B. contaminans* VC19124 | CF isolate, British Columbia (Canada) | CBCCRRR |  |
|  | *B. contaminans* VC9624 | CF isolate, Ontario (Canada) | CBCCRRR |  |
| ***Burkholderia diffusa*** | | |  | |
|  | *B. diffusa* HI2617 | Soil, celery field, New York (USA) | BcRLR |  |
|  | *B. diffusa* HI3576 | Soil, North Carolina (USA) | BcRLR |  |
|  | *B. diffusa* HI3672 | Soil, North Carolina (USA) | BcRLR |  |
|  | *B. diffusa* HI3740 | Soil, Michigan (USA) | BcRLR |  |
|  | *B. diffusa* VC14008 | CF isolate, Ontario (Canada) | CBCCRRR |  |
|  | *B. diffusa* VC15063 | CF isolate, Quebec (Canada) | CBCCRRR |  |
|  | *B. diffusa* VC6752 | CF isolate, Alberta (Canada) | CBCCRRR |  |
|  | *B. diffusa* VC6966 | Non-CF isolate, Quebec (Canada) | CBCCRRR |  |
|  | *B. diffusa* VC7394 | CF isolate, Quebec (Canada) | CBCCRRR |  |
|  | *B. diffusa* VC7913 | CF isolate, Quebec (Canada) | CBCCRRR |  |
| ***Burkholderia dolosa*** | | |  | |
|  | *B. dolosa* CEP0021 | CF isolate (Canada) | CBCCRRR |  |
|  | *B. dolosa* LMG21443 | *Alysicarpus glumaceus* root nodule | (12) |  |
|  | *B. dolosa* VC14902 | CF isolate, Alberta (Canada) | CBCCRRR |  |
|  | *B. dolosa* VC17647 | CF isolate, Quebec (Canada) | CBCCRRR |  |
| ***Burkholderia lata*** | | |  | |
|  | *B. lata* BC01 | River water, South Carolina (USA) | BcRLR |  |
|  | *B. lata* VC19230 | Clinical isolate, British Columbia (Canada) | CBCCRRR |  |
|  | *B. lata* VC6377 | CF isolate, Ontario (Canada) | CBCCRRR |  |
|  | *B. lata* VC8171 | CF isolate, New Brunswick (Canada) | CBCCRRR |  |
| ***Burkholderia metallica*** | | |  | |
|  | *B. metallica* ES0559 | Environmental isolate, Oregon (USA) | BcRLR |  |
|  | *B. metallica* HI3647 | Soil, North Carolina (USA) | BcRLR |  |
|  | *B. metallica* VC15467 | CF isolate, Ontario (Canada) | CBCCRRR |  |
|  | *B. metallica* VC8135 | Clinical isolate, British Columbia (Canada) | CBCCRRR |  |
| ***Burkholderia multivorans*** | | |  | |
|  | *B. multivorans* LMG16660 | CF isolate isolate (Canada) | CBCCRRR | CEP0781 |
|  | *B. multivorans* HI2790 | Doctor office (USA) | BcRLR |  |
|  | *B. multivorans* LMG17588 | Soil (USA) | (9) | ATCC17616/CEP0144 |
|  | *B. multivorans* VC12152 | Clinical isolate, British Columbia (Canada) | CBCCRRR |  |
|  | *B. multivorans* VC12258 | CF isolate, Saskatchewan (Canada) | CBCCRRR |  |
|  | *B. multivorans* VC12539 | CF isolate, British Columbia (Canada) | CBCCRRR |  |
|  | *B. multivorans* VC12675 | Clinical isolate, British Columbia (Canada) | CBCCRRR |  |
|  | *B. multivorans* VC13125 | CF isolate, British Columbia (Canada) | CBCCRRR |  |
|  | *B. multivorans* VC13145 | CF isolate, British Columbia (Canada) | CBCCRRR |  |
|  | *B. multivorans* VC13162 | CF isolate, Ontario (Canada) | CBCCRRR |  |
|  | *B. multivorans* VC13451 | Non-CF isolate, Nova Scotia (Canada) | CBCCRRR |  |
|  | *B. multivorans* VC13673 | CF isolate, Alberta (Canada) | CBCCRRR |  |
|  | *B. multivorans* VC13702 | CF isolate, Alberta (Canada) | CBCCRRR |  |
|  | *B. multivorans* VC13776 | CF isolate, Quebec (Canada) | CBCCRRR |  |
|  | *B. multivorans* VC14090 | CF isolate, Quebec (Canada) | CBCCRRR |  |
|  | *B. multivorans* VC14422 | NON CF isolate, Saskatchewan (Canada) | CBCCRRR |  |
|  | *B. multivorans* VC14443 | CF isolate, British Columbia (Canada) | CBCCRRR |  |
|  | *B. multivorans* VC14749 | CF isolate, British Columbia (Canada) | CBCCRRR |  |
|  | *B. multivorans* VC14757 | CF isolate, British Columbia (Canada) | CBCCRRR |  |
|  | *B. multivorans* VC15002 | CF isolate, Quebec (Canada) | CBCCRRR |  |
|  | *B. multivorans* VC15085 | CF isolate, Quebec (Canada) | CBCCRRR |  |
|  | *B. multivorans* VC15268 | CF isolate, British Columbia (Canada) | CBCCRRR |  |
|  | *B. multivorans* VC15273 | CF isolate, Ontario (Canada) | CBCCRRR |  |
|  | *B. multivorans* VC15814 | CF isolate, British Columbia (Canada) | CBCCRRR |  |
|  | *B. multivorans* VC15834 | CF isolate, Saskatchewan (Canada) | CBCCRRR |  |
|  | *B. multivorans* VC15873 | CF isolate, Quebec (Canada) | CBCCRRR |  |
|  | *B. multivorans* VC15952 | CF isolate, Quebec (Canada) | CBCCRRR |  |
|  | *B. multivorans* VC15953 | CF isolate, Ontario (Canada) | CBCCRRR |  |
|  | *B. multivorans* VC15977 | CF isolate, Alberta (Canada) | CBCCRRR |  |
|  | *B. multivorans* VC16475 | CF isolate, Quebec (Canada) | CBCCRRR |  |
|  | *B. multivorans* VC16487 | CF isolate, Quebec (Canada) | CBCCRRR |  |
|  | *B. multivorans* VC16759 | CF isolate, British Columbia (Canada) | CBCCRRR |  |
|  | *B. multivorans* VC16959 | CF isolate, British Columbia (Canada) | CBCCRRR |  |
|  | *B. multivorans* VC17546 | Clinical isolate, Saskatchewan (Canada) | CBCCRRR |  |
|  | *B. multivorans* VC18625 | CF isolate, Newfoundland and Labrador (Canada) | CBCCRRR |  |
|  | *B. multivorans* VC3419 | CF isolate, British Columbia (Canada) | CBCCRRR |  |
|  | *B. multivorans* VC4282 | CF isolate, British Columbia (Canada) | CBCCRRR |  |
|  | *B. multivorans* VC6534 | CF isolate, Quebec (Canada) | CBCCRRR |  |
|  | *B. multivorans* VC6564 | CF isolate, British Columbia (Canada) | CBCCRRR |  |
|  | *B. multivorans* VC7102 | CF isolate, British Columbia (Canada) | CBCCRRR |  |
|  | *B. multivorans* VC7704 | CF isolate, British Columbia (Canada) | CBCCRRR |  |
|  | *B. multivorans* VC7870 | CF isolate, Quebec (Canada) | CBCCRRR |  |
|  | *B. multivorans* VC7960 | CF isolate, British Columbia (Canada) | CBCCRRR |  |
|  | *B. multivorans* VC9159 | CF isolate, British Columbia (Canada) | BcRLR |  |
|  | *B. multivorans* VC9858 | CF isolate, British Columbia (Canada) | CBCCRRR |  |
| ***Burkholderia pyrrocinia*** | | |  | |
|  | *B. pyrrocinia* CH-67 | Soil, rhizosphere (Korea) | (13) |  |
|  | *B. pyrrocinia* ES0490 | Environmental isolate, Ohio (USA) | BcRLR | Bcc indeterminate 1 ES0490 |
|  | *B. pyrrocinia* BC02 | River water, South Carolina (USA) | BcRLR | Bcc indeterminate 2 BC02 |
|  | *B. pyrrocinia* HI2575 | Soil, New York (USA) | BcRLR | Bcc indeterminate 5 HI2575 |
|  | *B. pyrrocinia* HI2690 | Soil, rice field, New York (USA) | BcRLR | Bcc indeterminate 5 HI2690 |
|  | *B. pyrrocinia* HI2701 | Soil, New York (USA) | BcRLR | Bcc indeterminate 5 HI2701 |
|  | *B. pyrrocinia* HI3892 | Soil, Illinois (USA) | BcRLR | Bcc indeterminate 5 HI3892 |
|  | *B. pyrrocinia* ES0209 | Environmental isolate, Pennsylvania (USA) | BcRLR | Bcc indeterminate 9 ES0209 |
|  | *B. pyrrocinia* LMG21824 | CF isolate (UK) | (4) |  |
| ***Burkholderia* *seminalis*** | | |  | |
|  | *B. seminalis* HI2490 | Soil, New York (USA) | BcRLR |  |
| ***Burkholderia stabilis*** | | |  | |
|  | *B. stabilis* C7322 | CF isolate (Canada) | (14) |  |
|  | *B. stabilis* HI2462 | Contaminated shampoo (USA) | BcRLR |  |
|  | *B. stabilis* VC10097 | Non-CF isolate, Quebec (Canada) | CBCCRRR |  |
|  | *B. stabilis* VC12344 | CF isolate, Quebec (Canada) | CBCCRRR |  |
|  | *B. stabilis* VC12965 | Environmental isolate, Ontario (Canada) | CBCCRRR |  |
|  | *B. stabilis* VC17755 | Cancer, Alberta (Canada) | CBCCRRR |  |
|  | *B. stabilis* VC6296 | CF isolate, Alberta (Canada) | CBCCRRR |  |
|  | *B. stabilis* VC6482 | CF isolate, British Columbia (Canada) | CBCCRRR |  |
|  | *B. stabilis* VC6747 | CF isolate, British Columbia (Canada) | CBCCRRR |  |
|  | *B. stabilis* VC6749 | CF isolate, British Columbia (Canada) | CBCCRRR |  |
|  | *B. stabilis* VC6753 | CF isolate, British Columbia (Canada) | CBCCRRR |  |
|  | *B. stabilis* VC8622 | CF isolate, British Columbia (Canada) | CBCCRRR |  |
|  | *B. stabilis* VC8623 | CF isolate, British Columbia (Canada) | CBCCRRR |  |
|  | *B. stabilis* VC8629 | CF isolate, British Columbia (Canada) | CBCCRRR |  |
|  | *B. stabilis* VC8636 | CF isolate, British Columbia (Canada) | CBCCRRR |  |
|  | *B. stabilis* VC8638 | CF isolate, British Columbia (Canada) | CBCCRRR |  |
|  | *B. stabilis* VC8967 | Non-CF isolate, Ontario (Canada) | CBCCRRR |  |
|  | *B. stabilis* VC8971 | CF isolate, Ontario (Canada) | CBCCRRR |  |
|  | *B. stabilis* VC9042 | CF isolate, British Columbia (Canada) | CBCCRRR |  |
|  | *B. stabilis* VC9562 | CF isolate, Ontario (Canada) | CBCCRRR |  |
|  | *B. stabilis* VC9945 | CF isolate, Quebec (Canada) | CBCCRRR |  |
| ***Burkholderia stagnalis*** | | |  | |
|  | *B. stagnalis* HI2720 | Soil, tabasco, (Mexico) | BcRLR |  |
|  | *B. stagnalis* HI3537 | Soil, North Carolina (USA) | BcRLR | Bcc indeterminate 6 HI3537 |
|  | *B. stagnalis* MSMB1956WGS | Environmental isolate, North Carolina (USA) | MSHR-NAU |  |
| ***Burkholderia territorii*** | | |  | |
|  | *B. territorii* MSMB1301WGS | Environmental isolate, North Carolina (USA) | MSHR-NAU |  |
|  | *B. territorii* MSMB1502WGS | Environmental isolate, North Carolina (USA) | MSHR-NAU |  |
| ***Burkholderia ubonensis*** | | |  | |
|  | *B. ubonensis* LMG20358 | Environmental isolate (Thailand) | (2) | BCC1603 |
|  | *B. ubonensis* LMG24263 | Nosocomial (Thailand) | (15) |  |
| ***Burkholderia vietnamiensis*** | | |  | |
|  | *B. vietnamiensis* CEP0040 | CF isolate (Canada) | Mahenthiraligham collection | LMG 18835 |
|  | *B. vietnamiensis* G4 | Trichloroethene enrichment | (16) |  |
|  | *B. vietnamiensis* HI3534 | Barium, Texas (USA) | BcRLR |  |
|  | *B. vietnamiensis* VC0024 | CF isolate, British Columbia (Canada) | CBCCRRR |  |
|  | *B. vietnamiensis* VC10362 | CF isolate, British Columbia (Canada) | CBCCRRR |  |
|  | *B. vietnamiensis* VC10442 | Non-CF isolate, British Columbia (Canada) | CBCCRRR |  |
|  | *B. vietnamiensis* VC10676 | CF isolate, Quebec (Canada) | CBCCRRR |  |
|  | *B. vietnamiensis* VC11253 | CF isolate, British Columbia (Canada) | CBCCRRR |  |
|  | *B. vietnamiensis* VC11275 | CF isolate, British Columbia (Canada) | CBCCRRR |  |
|  | *B. vietnamiensis* VC11668 | CF isolate, Quebec (Canada) | CBCCRRR |  |
|  | *B. vietnamiensis* VC12002 | CF isolate, British Columbia (Canada) | CBCCRRR |  |
|  | *B. vietnamiensis* VC13138 | CF isolate, Quebec (Canada) | CBCCRRR |  |
|  | *B. vietnamiensis* VC13308 | CF isolate, Alberta (Canada) | CBCCRRR |  |
|  | *B. vietnamiensis* VC13830 | CF isolate, Quebec (Canada) | CBCCRRR |  |
|  | *B. vietnamiensis* VC13984 | CF isolate, Ontario (Canada) | CBCCRRR |  |
|  | *B. vietnamiensis* VC14091 | CF isolate, Quebec (Canada) | CBCCRRR |  |
|  | *B. vietnamiensis* VC14473 | CF isolate, Quebec (Canada) | CBCCRRR |  |
|  | *B. vietnamiensis* VC14737 | CF isolate, Quebec (Canada) | CBCCRRR |  |
|  | *B. vietnamiensis* VC15208 | CF isolate, British Columbia (Canada) | CBCCRRR |  |
|  | *B. vietnamiensis* VC15292 | CF isolate, Quebec (Canada) | CBCCRRR |  |
|  | *B. vietnamiensis* VC15774 | CF isolate, British Columbia (Canada) | CBCCRRR |  |
|  | *B. vietnamiensis* VC16431 | CF isolate, British Columbia (Canada) | CBCCRRR |  |
|  | *B. vietnamiensis* VC17180 | CF isolate, Quebec (Canada) | CBCCRRR |  |
|  | *B. vietnamiensis* VC17270 | CF isolate, Alberta (Canada) | CBCCRRR |  |
|  | *B. vietnamiensis* VC17399 | CF isolate, Quebec (Canada) | CBCCRRR |  |
|  | *B. vietnamiensis* VC17834 | CF isolate, Newfoundland and Labrador (Canada) | CBCCRRR |  |
|  | *B. vietnamiensis* VC18210 | CF isolate, British Columbia (Canada) | CBCCRRR |  |
|  | *B. vietnamiensis* VC18530 | CF isolate, Newfoundland and Labrador (Canada) | CBCCRRR |  |
|  | *B. vietnamiensis* VC18712 | CF isolate, Alberta (Canada) | CBCCRRR |  |
|  | *B. vietnamiensis* VC18844 | CF isolate, Quebec (Canada) | CBCCRRR |  |
|  | *B. vietnamiensis* VC2824 | CF isolate, British Columbia (Canada) | CBCCRRR |  |
|  | *B. vietnamiensis* VC5914 | CF isolate, Ontario (Canada) | CBCCRRR |  |
|  | *B. vietnamiensis* VC8245 | CF isolate, British Columbia (Canada) | CBCCRRR |  |
|  | *B. vietnamiensis* VC8613 | CF isolate, Alberta (Canada) | CBCCRRR |  |
|  | *B. vietnamiensis* VC9237 | CF isolate, British Columbia (Canada) | CBCCRRR |  |
|  | *B. vietnamiensis* VC9752 | CF isolate, British Columbia (Canada) | CBCCRRR |  |
| **Other Bcc group** | | |  | |
|  | *B.* sp LMI-SB2 |  |  |  |
|  | *B.* sp VC14128 | CF isolate, British Columbia (Canada) | CBCCRRR |  |
|  | *B.* sp VC15804 | CF isolate, Ontario (Canada) | CBCCRRR |  |
|  | *B.* sp VC16512 | Clinical isolate, Alberta (Canada) | CBCCRRR |  |
|  | *B.* sp VC18848 | CF isolate, Quebec (Canada) | CBCCRRR |  |
|  | Other Bcc - Bcc indeterminate 1 BC06 | River water, South Carolina (USA) | BcRLR |  |
|  | Other Bcc - Bcc indeterminate 3 BC13 | River water, South Carolina (USA) | BcRLR |  |
|  | Other Bcc - Bcc indeterminate 3 ES0139 | Environmental isolate, Pennsylvania (USA) | BcRLR |  |
|  | Other Bcc - Bcc indeterminate 4 BC04 | River water, South Carolina (USA) | BcRLR |  |
|  | Other Bcc - Bcc indeterminate 5 BC03 | River water, South Carolina (USA) | BcRLR |  |
|  | Other Bcc - Bcc indeterminate 8 HI4407 | Water, Kentucky (USA) | BcRLR |  |

CBCCRRR: Strains were provided by Canadian *Burkholderia cepacia* complex Research and Referral Repository, University of British Colombia, Canada

BcRLR: *Burkholderia cepacia* Research Laboratory and Repository, University of Michigan

MSHR-NAU: Menzies School of Health Research and Northern Arizona University, USA

**Table S2. Comparison of *in silico* and *in vitro* results of the distribution of the *hmqABCDEFG* operon**

| **Strains** | **Prevalence of *hmqABCDEFG* operon (%)** | |  |
| --- | --- | --- | --- |
|  | **Bioinformatics analysis by homology [total genome sequences]** | **Experimental analysis by PCR [total screened strains]** |  |
|  |  |  |  |
| ***B. cepacia* (genomovar I)** | 23 [337] | 90 [31] |  |
| ***B. multivorans* (genomovar II)** | 0 [56] | 0 [45] |  |
| ***B. cenocepacia* (genomovar III)** | 0 [243] | 0 [72] |  |
| ***B. stabilis* (genomovar IV)** | - | 0 [21] |  |
| ***B. vietnamiensis* (genomovar V)** | 0 [41] | 13 [36] |  |
| ***B. dolosa* (genomovar VI)** | 0 [2] | 25 [4] |  |
| ***B. ambifaria* (genomovar VII)** | 50 [6] | 68 [32] |  |
| ***B. anthina* (genomovar VIII)** | 0 [8] | 25 [4] |  |
| ***B. pyrrocinia* (genomovar IX)** | 75 [4] | 78 [9] |  |
| ***B. ubonensis* (genomovar X)** | 97 [292] | 50 [2] |  |
| ***B. latens* (BCC1)** | 0 [2] | - |  |
| ***B. diffusa* (BCC 2)** | 0 [12] | 10 [10] |  |
| ***B. arboris* (BCC 3)** | - | 0 [4] |  |
| ***B. seminalis* (BCC 7)** | 0 [3] | 100 [1] |  |
| ***B. metallica* (BCC 8)** | 0 [1] | 25 [4] |  |
| ***B. lata* (group K)** | 50 [4] | 50 [4] |  |
| ***B. contaminans* (group K, BCCAT)** | 43 [7] | 82 [17] |  |
| ***B. pseudomultivorans*** | 0 [9] | - |  |
| ***B. stagnalis* (BCC B)** | 98 [64] | 100 [3] |  |
| ***B. territorii* (BCC I)** | 6 [33] | 100 [2] |  |
| ***B. paludis*** | - | - |  |
| **Other Bcc group** | 18 [59] | 45 [11] |  |
| **Total** | 35 [1257] | 30 [312] |  |

Not available data are represented by “-“

Kendall's rank test with a p-value of 0.01958

**Table S3. Study of the presence of the third replicon in Bcc strains carrying or not the *hmqABCDEFG* operon**

| **Strains** | **Number of chromosomes** | **Presence of the *hmqABCDEFG* operon** |
| --- | --- | --- |
| *Burkholderia ambifaria* AMMD | 3 | + |
| *Burkholderia ambifaria* MC40-6 | 3 | + |
| *Burkholderia cenocepacia 842* | 3 | - |
| *Burkholderia cenocepacia* 895 | 2 | - |
| *Burkholderia cenocepacia* AU 1054 | 3 | - |
| *Burkholderia cenocepacia* CR318 | 3 | - |
| *Burkholderia cenocepacia* DDS 22E-1 | 3 | - |
| *Burkholderia cenocepacia* DWS 37E-2 | 3 | - |
| *Burkholderia cenocepacia* H111 | 3 | - |
| *Burkholderia cenocepacia* HI2424 | 3 | - |
| *Burkholderia cenocepacia* J2315 | 3 | - |
| *Burkholderia cenocepacia* MC0-3 | 3 | - |
| *Burkholderia cenocepacia* MSMB384WGS | 3 | - |
| *Burkholderia cenocepacia* VC12308 | 3 | - |
| *Burkholderia cenocepacia* VC12802 | 2 | - |
| *Burkholderia cenocepacia* VC7848 | 1 | - |
| *Burkholderia cepacia* ATCC 25416 | 3 | + |
| *Burkholderia cepacia* DDS 7H-2 | 3 | - |
| *Burkholderia cepacia* FDAARGOS_345 | 3 | + |
| *Burkholderia cepacia* FDAARGOS_388 | 3 | + |
| *Burkholderia cepacia* GG4 | 2 | - |
| *Burkholderia cepacia* INT3-BP177 | 2 | - |
| *Burkholderia cepacia* JBK9 | 3 | - |
| *Burkholderia cepacia* LO6 | 1 | - |
| *Burkholderia cepacia* MSMB1184WGS | 3 | + (c2) |
| *Burkholderia contaminans* MS14 | 3 | + |
| *Burkholderia diffusa* RF2-non-BP9 | 3 | - |
| *Burkholderia dolosa* AU0158 | 3 | - |
| *Burkholderia lata* 383 | 3 | - |
| *Burkholderia lata* FL-7-5-30-S1-D0 | 3 | + |
| *Burkholderia latens* AU17928 | 3 | - |
| *Burkholderia metallica* FL-6-5-30-S1-D7 | 3 | - |
| *Burkholderia multivorans* ATCC 17616 | 3 | - |
| *Burkholderia multivorans* ATCC 17616 | 3 | - |
| *Burkholderia multivorans* ATCC BAA-247 | 3 | - |
| *Burkholderia multivorans* AU1185 | 3 | - |
| *Burkholderia multivorans* MSMB1640WGS | 3 | - |
| *Burkholderia pyrrocinia* DSM 10685 | 3 | + |
| *Burkholderia seminalis* FL-5-4-10-S1-D7 | 3 | - |
| *Burkholderia stabilis* ATCC BAA-67 | 3 | - |
| *Burkholderia stabilis* FERMP-21014 | 3 | - |
| *Burkholderia stagnalis* MSMB735WGS | 3 | - |
| *Burkholderia territorii* RF8-non-BP5 | 3 | - |
| *Burkholderia ubonensis* MSMB0783 | 3 | - |
| *Burkholderia ubonensis* MSMB1189WGS | 3 | + |
| *Burkholderia ubonensis* MSMB1471WGS | 2 | + |
| *Burkholderia ubonensis* MSMB2035 | 3 | + |
| *Burkholderia ubonensis* MSMB22 | 3 | + |
| *Burkholderia ubonensis* RF23-BP41 | 3 | + |
| *Burkholderia vietnamiensis* AU1233 | 2 | - |
| *Burkholderia vietnamiensis* FL-2-3-30-S1-D0 | 3 | - |
| *Burkholderia vietnamiensis* G4 | 3 | - |
| *Burkholderia vietnamiensis* HI2297 | 3 | - |
| *Burkholderia vietnamiensis* LMG 10929 | 3 | - |
| *Burkholderia vietnamiensis* MSMB608WGS | 3 | - |

**Table S4. HMAQ production in different culture media at 30°C for strains having the *hmqABCDEFG* operon in their genome**

| **Strains** | **Type** | ***hmq* operon** | | **HMAQ production** | | |
| --- | --- | --- | --- | --- | --- | --- |
|  |  | ***hmqA*** | ***hmqG*** | **TSB** | **ASM** | **TSA** |
| *B. ambifaria* AMMD | Environmental | + | + | - | + | + |
| *B. ambifaria* AU0212 | Clinical | + | + | + | + | + |
| *B. ambifaria* AU4157 | Clinical | + | + | + | + | - |
| *B. ambifaria* AU7994 | Clinical | + | + | - | - | - |
| *B. ambifaria* CEP0617 | Clinical | + | + | + | + | - |
| *B. ambifaria* CEP0958 | Clinical | + | + | + | + | + |
| *B. ambifaria* CEP0990 | Clinical | + | + | + | - | - |
| *B. ambifaria* CEP0996 | Clinical | + | + | + | + | + |
| *B. ambifaria* CEP1231 | Clinical | + | + | - | + | - |
| *B. ambifaria* ES0020 | Environmental | + | + | + | + | + |
| *B. ambifaria* HI2425 | Environmental | + | + | - | + | + |
| *B. ambifaria* HI2468 | Environmental | + | + | + | + | + |
| *B. ambifaria* HI2482 | Environmental | + | + | + | + | + |
| *B. ambifaria* HI2626 | Environmental | + | + | + | + | + |
| *B. ambifaria* HI2672 | Environmental | + | + | + | + | + |
| *B. ambifaria* HI3709 | Environmental | + | + | + | + | + |
| *B. ambifaria* HI3738 | Environmental | + | + | + | + | + |
| *B. ambifaria* HSJ1 | Clinical | + | + | + | + | + |
| *B. ambifaria* PC736 | Environmental | + | + | + | + | + |
| *B. ambifaria* VC11631 | Clinical | + | + | - | - | - |
| *B. ambifaria* VC15422 | Clinical | + | + | + | + | + |
| *B. ambifaria* VC16196 | Clinical | + | + | + | + | + |
| *B. anthina* VC15382 | Clinical | + | + | - | - | - |
| *B. cepacia* BTS13 | Clinical | + | + | + | - | - |
| *B. cepacia* HI2430 | Environmental | + | + | + | + | + |
| *B. cepacia* HI2563 | Environmental | + | + | + | + | + |
| *B. cepacia* HI2578 | Environmental | + | + | + | + | + |
| *B. cepacia* HI2615 | Environmental | + | + | + | + | + |
| *B. cepacia* HI2671 | Environmental | + | + | + | + | + |
| *B. cepacia* HI2741 | Environmental | + | + | + | + | + |
| *B. cepacia* HI3312 | Environmental | + | + | + | + | + |
| *B. cepacia* HI3551 | Environmental | + | + | + | + | + |
| *B. cepacia* HI3708 | Environmental | + | + | + | + | + |
| *B. cepacia* HI3895 | Environmental | + | + | + | + | + |
| *B. cepacia* HI4577 | Environmental | + | + | + | - | + |
| *B. cepacia* ATCC25416 | Environmental | + | + | - | - | - |
| *B. cepacia* VC13132 | Clinical | + | + | + | + | + |
| *B. cepacia* VC13196 | Clinical | + | + | - | - | - |
| *B. cepacia* VC13394 | Clinical | + | + | + | + | + |
| *B. cepacia* VC13575 | Clinical | + | + | - | - | - |
| *B. cepacia* VC14106 | Clinical | + | + | + | + | + |
| *B. cepacia* VC14457 | Clinical | + | + | + | + | + |
| *B. cepacia* VC17333 | Clinical | + | + | + | + | + |
| *B. cepacia* VC17746 | Clinical | + | + | + | + | + |
| *B. cepacia* VC17928 | Clinical | + | + | + | + | + |
| *B. cepacia* VC18315 | Clinical | + | + | + | + | + |
| *B. cepacia* VC18839 | Clinical | + | + | + | + | + |
| *B. cepacia* VC18842 | Clinical | + | + | + | + | + |
| *B. cepacia* VC19225 | Clinical | + | + | - | + | + |
| *B. cepacia* VC19276 | Clinical | + | + | + | + | + |
| *B. cepacia* VC9490 | Clinical | + | + | + | + | + |
| *B. contaminans* FFH2055 | Clinical | + | + | + | - | - |
| *B. contaminans* HI3570 | Environmental | + | + | + | + | + |
| *B. contaminans* HI3852 | Environmental | + | + | + | + | + |
| *B. contaminans* HI4067 | Environmental | + | + | + | + | + |
| *B. contaminans* HI4232 | Environmental | + | + | + | + | + |
| *B. contaminans* VC14347 | Clinical | + | + | + | + | + |
| *B. contaminans* VC15406 | Clinical | + | + | - | - | + |
| *B. contaminans* VC16087 | Clinical | + | + | + | + | + |
| *B. contaminans* VC16848-b | Clinical | + | + | + | + | + |
| *B. contaminans* VC16897 | Clinical | + | + | - | - | - |
| *B. contaminans* VC16948 | Clinical | + | + | - | - | + |
| *B. contaminans* VC19056 | Clinical | + | + | + | + | + |
| *B. contaminans* VC19124 | Clinical | + | + | + | + | + |
| *B. contaminans* VC9624 | Clinical | + | + | + | + | + |
| *B. diffusa* VC14008 | Clinical | + | + | - | - | - |
| *B. dolosa* LMG21443 | Environmental | + | + | - | - | - |
| *B. lata* BC01 | Environmental | + | + | + | + | + |
| *B. lata* VC6377 | Clinical | + | + | - | + | + |
| *B. metallica* ES0559 | Environmental | + | + | - | - | - |
| *B. pyrrocinia* Bcc indeterminate 2 BC02 | Environmental | + | + | + | + | - |
| *B. pyrrocinia* Bcc indeterminate 5 HI2575 | Environmental | + | + | + | + | + |
| *B. pyrrocinia* Bcc indeterminate 5 HI2690 | Environmental | + | + | + | + | + |
| *B. pyrrocinia* Bcc indeterminate 5 HI2701 | Environmental | + | + | + | + | + |
| *B. pyrrocinia* Bcc indeterminate 5 HI3892 | Environmental | + | + | + | + | + |
| *B. pyrrocinia* Bcc indeterminate 9 ES0209 | Environmental | + | + | - | + | + |
| *B. pyrrocinia* CH-67 (LMG14191) | Environmental | + | + | + | + | + |
| *B. seminalis* HI2490 | Environmental | + | + | - | + | - |
| *B. stagnalis* Bcc indeterminate 6 HI3537 | Environmental | + | + | - | + | + |
| *B. stagnalis* HI2720 | Environmental | + | + | - | + | - |
| *B. stagnalis* MSMB1956WGS | Environmental | + | + | + | + | - |
| *B. territorii* MSMB1301WGS | Environmental | + | + | + | + | + |
| *B. territorii* MSMB1502WGS | Environmental | + | + | + | + | + |
| *B. ubonensis* LMG24263 | Clinical | + | + | - | - | - |
| *B. vietnamiensis* CEP0040 | Clinical | + | + | - | - | - |
| *B. vietnamiensis* HI3392 | Environmental | + | + | - | + | + |
| *B. vietnamiensis* VC17180 | Clinical | + | + | - | - | - |
| *B. vietnamiensis* VC8245 | Clinical | + | + | + | + | + |
| *B. vietnamiensis* VC9237 | Clinical | + | + | - | - | - |
| other Bcc - Bcc indeterminate 1 BC06 | Environmental | + | + | + | + | + |
| other Bcc - Bcc indeterminate 3 BC13 | Environmental | + | + | + | + | + |
| other Bcc - Bcc indeterminate 3 ES0139 | Environmental | + | + | + | + | + |
| other Bcc - Bcc indeterminate 4 BC04 | Environmental | + | + | + | + | + |
| other Bcc - Bcc indeterminate 5 BC03 | Environmental | + | + | + | + | + |

**Table S5. Quantification of the production of HMAQs for the Bcc strains which do not carry the *hmqABCDEFG* operon in their genome**

| **Strains** | **Type** | ***hmqA*** | ***hmqG*** | **HMAQs production (TSB)** |
| --- | --- | --- | --- | --- |
| *B. ambifaria* CEP0516 | Clinical | **-** | **-** | **-** |
| *B. ambifaria* HI3590 | Environmental | **-** | **-** | **-** |
| *B. ambifaria* HI3687 | Environmental | **-** | **-** | **-** |
| *B. ambifaria* IOP40-10 | Environmental | **-** | **-** | **-** |
| *B. ambifaria* LMG17828 | Environmental | **-** | **-** | **-** |
| *B. ambifaria* PHP7 | Environmental | **-** | **-** | **-** |
| *B. anthina* VC16083 | Clinical | **-** | **-** | **-** |
| *B. arboris* VC8833 | Clinical | **-** | **-** | **-** |
| *B. cenocepacia* CEP024 | Clinical | **-** | **-** | **-** |
| *B. cenocepacia* CEP0511 | Clinical | **-** | **-** | **-** |
| *B. cenocepacia* CEP0565 | Clinical | **-** | **-** | **-** |
| *B. cenocepacia* IIIA VC16156 | Clinical | **-** | **-** | **-** |
| *B. cenocepacia* IIIB VC11311 | Clinical | **-** | **-** | **-** |
| *B. cenocepacia* IIIB VC15122 | Clinical | **-** | **-** | **-** |
| *B. cenocepacia* K56-2 | Clinical | **-** | **-** | **-** |
| *B. cenocepacia* LMG19240 | Environmental | **-** | **-** | **-** |
| *B. multivorans* CEP0781 | Clinical | **-** | **-** | **-** |
| *B. multivorans* LMG17588 | Environmental | **-** | **-** | **-** |
| *B. multivorans* VC14090 | Clinical | **-** | **-** | **-** |
| *B. multivorans* VC16759 | Clinical | **-** | **-** | **-** |
| *B. pyrrocinia* LMG21824 | Clinical | **-** | **-** | **-** |
| *B. stabilis* LMG18870 | Clinical | **-** | **-** | **-** |
| *B. stabilis* VC6749 | Clinical | **-** | **-** | **-** |
| *B. stabilis* VC6753 | Clinical | **-** | **-** | **-** |
| *B. ubonensis* LMG20358 | Environmental | **-** | **-** | **-** |
| B. vietnamiensis G4 | Environmental | **-** | **-** | **-** |
| *B. vietnamiensis* VC13984 | Clinical | **-** | **-** | **-** |
| *B. vietnamiensis* VC18210 | Clinical | **-** | **-** | **-** |
| *B. vietnamiensis* VC18712 | Clinical | **-** | **-** | **-** |
| *B. vietnamiensis* VC18844 | Clinical | **-** | **-** | **-** |
| *B. vietnamiensis* VC2824 | Clinical | **-** | **-** | **-** |

**Table S6. HMAQs and HAQs congeners produce by the Bcc strains**

HMAQs production are in ppm (mg/L).

**Table S7. Correlation data on the presence of the *hmqABCDEFG* operon and the production of HMAQs in clinical Bcc isolates.**

| **Species** | ***hmq* operon** | **HMAQ**  **production** | **Co-isolated with *Pseudomonas***  **+/- 7 days** | ***Pseudomonas* in the previous year** | **Source** |
| --- | --- | --- | --- | --- | --- |
| ***B. cenocepacia* IIIA VC12308** | no | no | yes | yes | sputum |
| ***B. cenocepacia* IIIA VC15419** | no | no | no | no | sputum |
| ***B. cenocepacia* IIIA VC18585** | no | no | yes | no | respiratory |
| ***B. cenocepacia* IIIA VC18996** | no | no | no | no | sputum |
| ***B. cenocepacia* IIIA VC18999** | no | no | no | no | throat |
| ***B. cenocepacia* IIIA VC3917** | no | no | yes | yes | sputum |
| ***B. cenocepacia* IIIA VC6356** | no | no | no | yes | sputum |
| ***B. cenocepacia* IIIA VC6553** | no | no | no | yes | sputum |
| ***B. cenocepacia* IIIB VC11311** | no | no | yes | yes | sputum |
| ***B. cenocepacia* IIIB VC15122** | no | no | no | no | throat |
| ***B. cenocepacia* IIIB VC6598** | no | no | yes | yes | sputum |
| ***B. cenocepacia* IIIB VC7349** | no | no | no | yes | respiratory |
| ***B. cenocepacia* IIIB VC7849** | no | no | no | yes | sputum |
| ***B. cenocepacia* IIIB VC7911** | no | no | no | no | respiratory |
| ***B. cenocepacia* IIIB VC8340** | no | no | yes | yes | respiratory |
| ***B. cepacia* VC18315** | yes | yes | no | yes | respiratory |
| ***B. cepacia* VC9490** | yes | yes | yes | no | sputum |
| ***B. contaminans* VC14347** | yes | yes | yes | yes | respiratory |
| ***B. contaminans* VC15406** | yes | no | no | yes | sinus |
| ***B. contaminans* VC16087** | yes | yes | no | no | respiratory |
| ***B. lata* VC19230** | no | no | no | no | throat |
| ***B. metallica* VC8135** | no | no | no | yes | sputum |
| ***B. multivorans* VC12152** | no | no | yes | no | respiratory |
| ***B. multivorans* VC12539** | no | no | no | yes | respiratory |
| ***B. multivorans* VC12675** | no | no | no | no | sputum |
| ***B. multivorans* VC13125** | no | no | no | no | respiratory |
| ***B. multivorans* VC13145** | no | no | no | no | sputum |
| ***B. multivorans* VC14443** | no | no | no | no | sputum |
| ***B. multivorans* VC14749** | no | no | no | yes | sputum |
| ***B. multivorans* VC14757** | no | no | no | yes | respiratory |
| ***B. multivorans* VC15814** | no | no | no | no | respiratory |
| ***B. multivorans* VC16959** | no | no | no | no | sputum |
| ***B. multivorans* VC3419** | no | no | yes | yes | sputum |
| ***B. multivorans* VC4282** | no | no | no | yes | sputum |
| ***B. multivorans* VC6564** | no | no | yes | yes | sputum |
| ***B. multivorans* VC7102** | no | no | yes | yes | throat |
| ***B. multivorans* VC7704** | no | no | yes | yes | sputum |
| ***B. multivorans* VC7960** | no | no | no | no | respiratory |
| ***B. multivorans* VC9159** | no | no | no | no | respiratory |
| ***B.* sp VC14128** | no | no | no | no | respiratory |
| ***B. stabilis* VC6482** | no | no | yes | yes | sputum |
| ***B. stabilis* VC9042** | no | no | no | no | respiratory |
| ***B. vietnamiensis* VC10362** | no | no | no | no | respiratory |
| ***B. vietnamiensis* VC11253** | no | no | no | no | sputum |
| ***B. vietnamiensis* VC11275** | no | no | no | yes | respiratory |
| ***B. vietnamiensis* VC12002** | no | no | no | no | respiratory |
| ***B. vietnamiensis* VC15208** | no | no | no | no | sputum |
| ***B. vietnamiensis* VC15774** | no | no | no | yes | sputum |
| ***B. vietnamiensis* VC16431** | no | no | no | no | respiratory |
| ***B. vietnamiensis* VC18210** | no | no | no | no | respiratory |
| ***B. vietnamiensis* VC2824** | no | no | yes | yes | sputum |
| ***B. vietnamiensis* VC9237** | yes | no | yes | yes | respiratory |
| ***B. vietnamiensis* VC9752** | no | no | no | no | respiratory |

**Table S8. Primers used in this study**

| **Name** | **Sequence** | **Function** | **Reference** |  |
| --- | --- | --- | --- | --- |
| hisA_Bcc_F | GGTCGACCTGAACGGCGC | Reference gene for PCR screening | (17) |  |
| hisA_Bcc_R | CGTCGGTCGCGACCTTGCC |  | (17) |  |
| hmqA_Bcc_F | CCGCTCGCGTT**Y**ACGTT**Y**GG | Amplification of *hmqA* gene in Bcc - degenerated | This study |  |
| hmqA_Bcc_R | CCCGTCAGGTTCCAGCCG |  | This study |  |
| hmqG_Bcc_F | GGCGTCGCAGGAAATCACG | Amplification of *hmqG* gene in Bcc - degenerated | This study |  |
| hmqG_Bcc_R | CGCGACACGAA**R**TGCATGCC |  | This study |  |
| recA_Bcc_F | GATAGCAAGAAGGGCTCC | Identification of Bcc | Pubmlst |  |
| recA_Bcc_R | CTCTTCTTCGTCCATCGCCTC |  | Pubmlst |  |
| gyrB_Bcc_F | CGACAACTCGATCGACGA |  | Pubmlst |  |
| gyrB_Bcc_R | GACAGCAGCTTGTCGTAG |  | Pubmlst |  |
| recA_Bcc_seq_F | TGACCGCCGAGAAGAGCAA | Sanger sequencing | Pubmlst |  |
| recA_Bcc_seqR | GACCGAGTCGATGACGAT |  | Pubmlst |  |
| gyrB_Bcc_seq_F | ATCGTGATGACCGAGCTG |  | Pubmlst |  |
| gyrB_Bcc_seqR | CGTTGTAGCTGTCGTTCC |  | Pubmlst |  |
| ndh_deb_F | GCTCGGCTACGACGATCT | Reference gene for RT-PCR | This study | |
| ndh_ deb_R | GGCCTGGTCGAGGTTTTC |  | This study |  |
| hmqA _RTPCR_F | CTTGCCCCCTGCCGAAGATT | Expression of *hmqA* by RT-PCR | This study |  |
| hmqA _RTPCR_R | CGGCGCAATTGAGAAACGG |  | This study |  |

**References**

1. Sievers F, Higgins DG. 2014. Clustal Omega, accurate alignment of very large numbers of sequences, p. 105–116. In Russell, DJ (ed.), Multiple Sequence Alignment Methods. Methods in Molecular Biology (Methods and Protocols). Humana Press, Totowa, NJ.

2. Coenye T, Vandamme P, Govan JRW, LiPuma JJ. 2001. Taxonomy and Identification of the *Burkholderia cepacia* Complex. J Clin Microbiol 39:3427–3436.

3. Payne GW, Vandamme P, Morgan SH, LiPuma JJ, Coenye T, Weightman AJ, Jones TH, Mahenthiralingam E. 2005. Development of a *recA* gene-based identification approach for the entire *Burkholderia* genus. Appl Environ Microb 71:3917–3927.

4. Coenye T, Vandamme P, LiPuma JJ, Govan JRW, Mahenthiralingam E. 2003. Updated version of the *Burkholderia cepacia* Complex experimental strain panel. J Clin Microbiol 41:2797–2798.

5. Vial L, Lépine F, Milot S, Groleau M-C, Dekimpe V, Woods DE, Déziel E. 2008. *Burkholderia pseudomallei, B. thailandensis*, and *B. ambifaria* produce 4-hydroxy-2-alkylquinoline analogues with a methyl group at the 3 position that is required for quorum-sensing regulation. J Bacteriol 190:5339–5352.

6. Baldwin A, Mahenthiralingam E, Thickett KM, Honeybourne D, Maiden MCJ, Govan JR, Speert DP, LiPuma JJ, Vandamme P, Dowson CG. 2005. Multilocus sequence typing scheme that provides both species and strain differentiation for the *Burkholderia cepacia* Complex. J Clin Microbiol 43:4665–4673.

7. LiPuma JJ, Spilker T, Gill LH, Campbell PW, Liu L, Mahenthiralingam E. 2001. Disproportionate distribution of Burkholderia cepacia complex species and transmissibility markers in cystic fibrosis. Am J Respir Crit 164:92–96.

8. Yabuuchi E, Kosako Y, Oyaizu H, Yano I, Hotta H, Hashimoto Y, Ezaki T, Arakawa M. 1992. Proposal of *Burkholderia* gen. nov. and transfer of seven species of the genus *Pseudomonas* homology group ii to the new genus, with the type species *Burkholderia cepacia* (Palleroni and Holmes 1981) comb. nov. Microbiol Immunol 36:1251–1275.

9. Vandamme P, Holmes B, Vancanneyt M, Coenye T, Hoste B, Coopman R, Revets H, Lauwers S, Gillis M, Kersters K, Govan JRW. 2008. Occurrence of multiple genomovars of *Burkholderia cepacia* in cystic fibrosis patients and proposal of *Burkholderia multivorans* sp. nov. Int J Syst Bacteriol 47:1188–1200.

10. Lagatolla C, Skerlavaj S, Dolzani L, Tonin EA, Bragadin CM, Bosco M, Rizzo R, Giglio L, Cescutti P. 2002. Microbiological characterisation of *Burkholderia cepacia* isolates from cystic fibrosis patients: investigation of the exopolysaccharides produced. Fems Microbiol Lett 209:99–106.

11. Nunvar J, Kalferstova L, Bloodworth RAM, Kolar M, Degrossi J, Lubovich S, Cardona ST, Drevinek P. 2016. Understanding the pathogenicity of *Burkholderia contaminans*, an emerging pathogen in cystic fibrosis. PLoS One 11:e0160975–19.

12. Vandamme P, Henry D, Coenye T, Nzula S, Vancanneyt M, LiPuma JJ, Speert DP, Govan JRW, Mahenthiralingam E. 2002. *Burkholderia anthina* sp. nov. and *Burkholderia pyrrocinia*, two additional *Burkholderia cepacia* complex bacteria, may confound results of new molecular diagnostic tools. FEMS Immunol Med Microbiol 33:143–149.

13. Lee KY, Kong HG, Choi KH, Lee SW, Moon BJ. 2011. Isolation and identification of *Burkholderia pyrrocinia* CH-67 to control tomato leaf mold and damping-off on crisphead lettuce and tomato. Plant Pathology J 27:59–67.

14. Mahenthiralingam E, Bischof J, Byrne SK, Radomski C, Davies JE, Av-Gay Y, Vandamme P. 2000. DNA-Based diagnostic approaches for identification of *Burkholderia cepacia* complex, *Burkholderia vietnamiensis*, *Burkholderia multivorans*, *Burkholderia stabilis*, and *Burkholderia cepacia* genomovars I and III. J Clin Microbiol 38:3165–3173.

15. Vanlaere E, Sergeant K, Dawyndt P, Kallow W, Erhard M, Sutton H, Dare D, Devreese B, Samyn B, Vandamme P. 2008. Matrix-assisted laser desorption ionisation-time-of of-flight mass spectrometry of intact cells allows rapid identification of *Burkholderia cepacia* complex. J Microbiol Meth 75:279–286.

16. Nelson MJ, Montgomery SO, Mahaffey WR, Pritchard PH. 1987. Biodegradation of trichloroethylene and involvement of an aromatic biodegradative pathway. Appl Environ Microb 53:949–54.

17. Papaleo MC, Perrin E, Maida I, Fondi M, Fani R, Vandamme P. 2010. Identification of species of the *Burkholderia cepacia* complex by sequence analysis of the *hisA* gene. J Med Microbiol 59:1163–70.
